# Learning the mechanisms of network growth

**DOI:** 10.1038/s41598-024-61940-4

**Published:** 2024-05-24

**Authors:** Lourens Touwen, Doina Bucur, Remco van der Hofstad, Alessandro Garavaglia, Nelly Litvak

**Affiliations:** 1 Department of Mathematics and Computer Science, Eindhoven University of Technology, Groene Loper 3, 5612 AE Eindhoven, The Netherlands; 2 Faculty of Electrical Engineering, Mathematics and Computer Science, University of Twente, Drienerlolaan 5, 7522 NB Enschede, The Netherlands

**Keywords:** Applied mathematics, Computer science, Scientific data, Statistical physics, thermodynamics and nonlinear dynamics

## Abstract

We propose a novel model-selection method for dynamic networks. Our approach involves training a classifier on a large body of synthetic network data. The data is generated by simulating nine state-of-the-art random graph models for dynamic networks, with parameter range chosen to ensure exponential growth of the network size in time. We design a conceptually novel type of dynamic features that count new links received by a group of vertices in a particular time interval. The proposed features are easy to compute, analytically tractable, and interpretable. Our approach achieves a near-perfect classification of synthetic networks, exceeding the state-of-the-art by a large margin. Applying our classification method to real-world citation networks gives credibility to the claims in the literature that models with preferential attachment, fitness and aging fit real-world citation networks best, although sometimes, the predicted model does not involve vertex fitness.

## Introduction

In this paper, we investigate model selection for dynamic growing networks using machine learning. By model selection we mean choosing a generative mathematical model that describes the dynamic network in the best possible way. We apply our methods to citation networks, as these are prominent examples of dynamic networks where vertices and edges remain in the network forever.

### Model selection for complex networks

In dynamic networks, model selection identifies generative *mechanisms* that drive the formation of new connections. There are numerous ways in which model selection can help solve network problems: (a) The specific topology of a graph affects the performance of algorithms for common tasks such as computing the shortest paths ^1,2^ and finding strongly connected components ^3^. Therefore, the classification of generative network-growth mechanisms of real-life networks is useful for selecting suitable network algorithms and forecasting their performance as well as their requirements for computing power and storage. (b) Scale-free networks with the same exponent but different degree cut-off values can be small- or ultra-small worlds ^4^. As such, the right model is necessary for accurate prediction of the shortest paths in networks. (c) Exact assumptions on the scale-free model may lead to the opposite conclusions on the epidemic threshold being positive ^5^ (the network has initial resistance to an epidemic) or zero ^6^ (even the smallest infection spreads widely). (d) Predictions of the network robustness depend on the fine details of the model. Indeed, a scale-free preferential attachment model of the Internet predicts an alarming sensitivity to targeted attacks ^7^, while more realistic models suggest a much higher robustness ^8^. (e) Different models assign different importance to the vertices. For example, PageRank follows a power-law distribution with the same exponent as the in-degree in a scale-free directed configuration model ^9^, but, surprisingly, has a smaller exponent, thus higher variability, in directed preferential attachment networks ^10^. (f) Last but not least, realistic models enable us to predict the growth and evolution of networks in the future.

The common approach for model selection is to fit the parameters of a generative model to the target network, generate synthetic networks with these fitted parameters, and compare their features (e.g., degree distributions, shortest paths, etc.) to the target network ^11–16^. However, fitting the model parameters is a difficult problem in itself, so only simple models admit explicit analytical results ^17,18^. Numerical methods for parameter fitting are available for a wider range of models, for instance,^19^ fits a discrete choice model, where the probability of an edge from *u* to *v* depends on the features (e.g., degrees) of *u* and *v*. The parameters are fitted using logit regression, where a large regression coefficient implies that the feature is important. The downside is that the model must be fitted for each network separately, and the parameter estimates are analytically intractable, thus conclusions do not easily generalize to other networks. Interestingly, experiments in^19^ find all features statistically significant, even when coefficients are small. This illustrates a fundamental challenge in model selection for complex networks, in that statistical tests are likely to reject any generative model.

Here we propose a machine learning approach to model selection that works as follows: First, many synthetic networks are generated from a particular set of generative models. Then a classifier is trained based on the network features. As a result, the classifier labels any network as being produced by one of the generative models. Recent work on machine learning for model selection usually compares fundamentally different models, such as Erdős-Rényi (ER) (random connections) vs. Barabási–Albert (BA) (connection probability to a vertex is proportional to its current degree) ^20^, or ER vs. BA vs. Chung-Lu (connection probability to a vertex is proportional to its fixed weight) vs. Hyperbolic Random Graphs (connection probability to a vertex is defined by its position in a hyperbolic space) ^21^. Some works also compare distinct models to real-life networks from different domains ^22,23^. In this work, we view a generative model as a combination of growth mechanisms. More specifically, we apply our methods for selecting the best combinations of growth mechanisms used in the literature to describe citation networks: fitness, aging, and preferential attachment ^19,24–28^.

An important contribution of this work is the novel feature design for dynamic networks. The literature mostly uses a set of features that in fact are metrics of the network’s final snapshot, for instance, the degree distribution, the PageRank distribution, the number of triangles, etc. ^15,16,21,22,29^ (see more details in “Machine learning methodology” section). There are also deep learning methods that take the entire adjacency matrix as input ^20,30^. We call these input features *static* because they do not explicitly include the network evolution. Instead, we propose new *dynamic* features inspired by the state-of-the-art network models^14,31^, and the growing data availability of dynamic networks (e.g., https://networkrepository.com/dynamic.php)^32^. Our features are aggregated statistics of the network growth in time. These statistics are interpretable, easy to compute, and have explicit analytical expressions suitable for mathematical derivations of their properties. Our method achieves almost perfect classification of synthetic data obtained from the state-of-the-art network models^14,31^. Our results on real-world citation data mostly support the current models^24–27^ with fitness, aging and preferential attachment as discussed next, but also show that the selected model may depend on a specific feature design, thus telling a cautionary tale for blanket application of these models as well as the usage of machine learning for model selection.

### Dynamic network mechanisms

We propose network models where edges do not disappear. Citation networks are a natural real-world example of such networks. Motivated by the properties of citation networks, our models include three generative mechanisms: fitness, aging and preferential attachment.

*Fitness* Some papers generate tremendous follow-up work, while others may not. One may say that in terms of attracting citations, not all papers are equally *fit*. Fitness is the mechanism that allows relatively young papers to be cited a lot. Wang, Song, and Barabási^24^ estimate the fitness of papers based on citations they receive. Zhou, Holme, Gong, et al.^28^ propose a model of link formation based on fitness and degrees. They find that, for citation networks like most non-social networks, the evolution based on fitness describes the degree distribution and the degree ratio of adjacent nodes better than the evolution based on degrees. Moreover, in models with fitness but without preferential attachment, power-law distribution of fitness results in power-law degree distribution (see Proposition A.1 in the Supplementary Material).

*Aging* Citations to a paper depend on the (calendar) time since its publication (see^33^ and Figure A.2b in the Supplementary Material). The literature suggests that aging occurs according to a log-normal distribution in calendar time (see^24^ and Figure A.3c in the Supplementary Material).

*Preferential Attachment* Highly-cited papers can be expected to get even more citations, thus giving rise to a preferential attachment effect. This effect was already observed in the context of citation networks by De Solla Price^34^, and was put forward as a main driver for the occurrence of power-law degrees in^11^. See e.g.,^24^, as well as^35^ for an attempt to measure this effect. Contrary to fitness, preferential attachment means that papers receive extra citations *because* they have already been cited, and not because they are fit. This effect may be due to scientists reading paper A that cites paper B, and thus citing B as well.

### Modeling networks in calendar time

Most models of dynamic networks take the *network growth time* approach, that is, vertices arrive one by one at time $t=1,2,\ldots$. We instead propose a continuous-time approach that mimics the networks’ exponential growth in *calendar time* (illustrated in Figure A.3a of the Supplementary Material). Our approach relies on the powerful concept of *continuous-time branching processes* (CTBP)^14,36,37^. We use CTBPs to produce random trees, and then transition from trees to graphs with random out-degrees using a *collapsing* procedure that identifies several vertices in the CTBP to one vertex in the random graph. We refer to Sect. 4.1 for details.

## Results

### Dynamic network models

**Figure 1:**
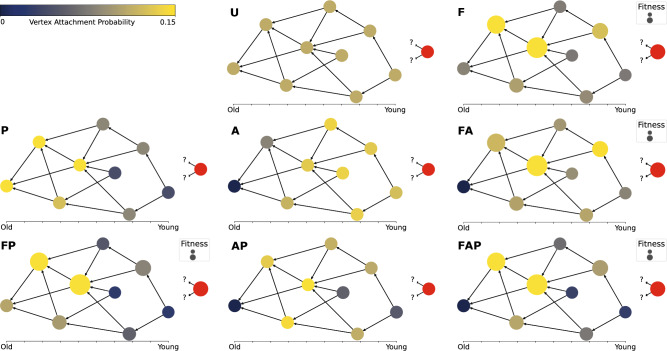
An example of the eight combinations of growth mechanisms. F stands for fitness, A for aging, and P for Preferential Attachment. U stands for uniform attachment, when a new vertex connects to existing ones uniformly at random. The circles are the vertices, arranged horizontally from left to right in the order of their arrival. The vertical positioning is chosen to make all directed edges visible, it has no further meaning. In the models with fitness, the size of the circles is proportional to their fitness. The red circle on the right is the new vertex that will connect to one of the existing vertices. The color of the other vertices from dark blue to yellow corresponds to the connection probability, from low to high, of the red vertex to an older vertex.

We model a network as a collapsed CTBP. By switching each of the generative mechanisms – fitness, aging, and preferential attachment – on or off, we obtain a flexible set of models that we wish to compare to real-world network data sets. Figure 1 illustrates the eight possible combinations of mechanisms. We use acronyms F (fitness), A (aging) and P (preferential attachment). The illustrations demonstrate how in the models with aging, the probability to connect to the older vertex is low; in the models with preferential attachment, the connection probability increases with degree; and in the models with fitness, the fit vertices have a higher connection probability. In addition to the eight different combinations of mechanisms, in models F and FA, we consider two distributions of fitness: power-law and exponential. We denote this by ${\hbox {F}_{\textsc {PL}}}$ and ${\hbox {F}_{\textsc {exp}}}$ , respectively. Model FP requires bounded fitness^38^, therefore we assume that the fitness has a uniform distribution and denote this by $\hbox {F}_\textsc {unif}$. Finally, we disregard model A (only aging) because it lacks the property of exponential growth in calendar time (see Sect. A.3 of the Supplementary Material). Altogether, we obtain a set of nine models from which we generate synthetic data to train our classifier for machine learned model selection. In the classification problem, each generative model corresponds to one category, or class.

The models proposed in this paper come with many parameters. We choose these parameters in such a way that the network characteristics obey asymptotic laws that are close to those observed in real-world networks. In particular, to mimic citation networks, we aim for models whose sizes grows exponentially in time. Mathematically, it means that the collapsed CTBP must satisfy a certain *supercriticality condition*, explained in Sect. A.3 of the Supplementary Material. Table 3 in Sect. 4 gives an overview of the models and the parameters used to generate the synthetic data set.

### Classification of dynamic networks

**Generating the training data.** For training, we have generated 6733 synthetic networks, about 750 for each of the nine generative models (categories), of size 20, 000. The numbers and size of synthetic networks are chosen so that the learning curves of machine learning models flatten at these data and network sizes (see Sect. A.6 in the Supplementary Material). Synthetic networks are generated by the following procedure. For a given generative model, the parameters are chosen uniformly at random from the range in Table 3. Then we let the network grow until it reaches 20, 000 vertices. If the CTBP dies out, we repeat the simulations with the same parameter combination at most 1000 times, until one of the runs reaches 20,000 vertices before dying out. The resulting network of 20, 000 vertices is included in the dataset. A few parameter combinations result is a very low survival probability, so we do not succeed in 1000 attempts; then we have slightly less than 750 synthetic networks of the corresponding category.

**Static features.** Static features use only the final snapshot of the graph. We use 36 static features from the recent literature^15,16,21,23,39^, that are feasible to compute on many medium-to-large size networks. They include assortativity, transitivity, and the distributions of numerical values associated with each vertex: degree, coreness, local clustering coefficient, and the number of triangles. See “Machine learning methodology” section for details.

**Dynamic features.** Our novel *dynamic* features significantly depart from the standard static features: they explicitly capture the network’s aggregated dynamics. The literature either learns the model from the final snapshots of many networks^15,16,21,23,39^ thus ignoring the dynamics, or considers each edge as a data point and learns the model from the set of edges of one network ^19^ thus allowing no generalization of the learned model to other networks. Our approach strikes the balance between viewing the network as a whole and at the same time including its dynamics. Specifically, denote the network graph at time $t\in [0,T]$ by $G(t)=(V(t), E(t))$, where *V*(*t*) is the set of vertices, and *E*(*t*) is the set of edges. We subdivide vertices in *V*(*T*) in equal groups $V_j$, $j\in \{1,2,\ldots , r\}$, by their degrees in the final graph *G*(*T*) from large to small. Furthermore, we subdivide the time interval *T* in *s* sub-intervals. We do this in two ways: using *size*-cohorts (where each sub-interval contains the same number of arrivals, |*V*(*T*)|/*s*), and using *time*-cohorts (where each sub-interval is of the same length, *T*/*s*, in calendar time). The dynamic features $D_{ij}$ are the average numbers of edges per vertex, received by group $V_j$ in the *i*-th sub-interval (see “Machine learning methodology” section  for the formal definition). This way the features $(D_{ij})_{1\le i\le s,1\le j\le r}$ track the network’s dynamics. We call $D=(D_{ij})$ the Dynamic Feature Matrix (DFM).

### Near-perfect classification of synthetic dynamic networks

**Figure 2:**
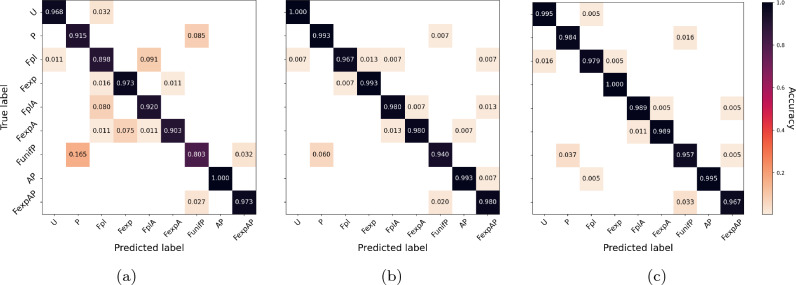
Confusion matrix using: (**a**) static features, (**b**) dynamic features with time cohorts, (**c**) both static features and dynamic features with time cohorts.

**Classification results with static features.** Static features already yield high accuracy of $92.81\pm 1\%$, where $92.81\%$ is the accuracy in the reported train-test split and 1% is the maximal observed absolute difference with other train-test splits in our experiments. The confusion matrix in Figure 2a predictably shows that the most common confusion is between P and F_unif_P. For discussion of feature importance, see Sect. A.8 and Figure A.7a in the Supplementary Material.

**Classification results with dynamic features.** Our dynamic features convincingly outperform static features, yielding near-perfect accuracy $98.06\pm 0.5$% with time-cohorts, and $97.62\pm 0.5\%$ with size-cohorts. The confusion matrix with time-cohorts in Figure 2b shows a significant improvement.

**Figure 3:**
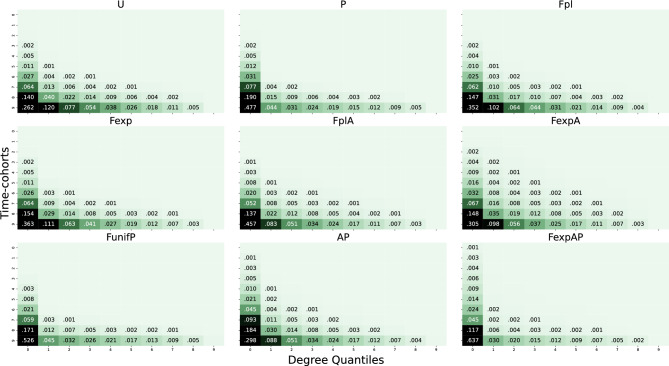
The values of the dynamic features with time-cohorts for the nine models. The shape of the matrix is similar for all models but the values reduce with their distance to $D_{10,1}$ in different ways.

The next natural question is, can we interpret the difference between generative models using the dynamic features? The standard permutation importance approach (see Sect. A.8 and Figure A.7b in the Supplementary Material) as well as extensive computations of correlations between features (omitted in this article, see Sect. A.12 in the Supplementary Material), did not offer any intelligible way to identify most informative features. Therefore, we propose a new approach to interpretation of the dynamic features. To begin with, Figure 3 shows the values of the dynamic features for the nine models.

**Figure 4:**
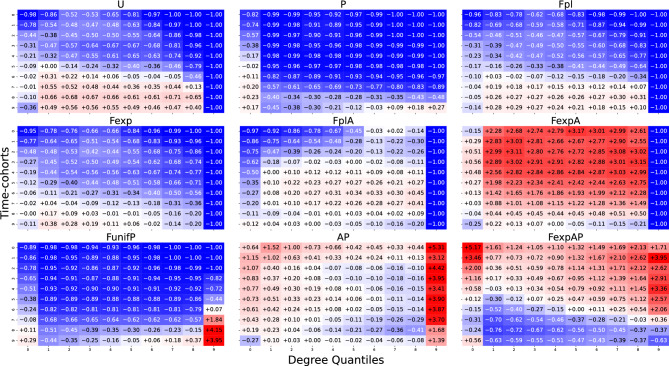
The relative difference, $\delta _{ij}=(D_{ij}-\bar{D}_{ij})/\bar{D}_{ij}$ with time-cohorts. The sum of elements *i*, *j* over all classes equals zero. The value $-1$ appears often because it corresponds to $D_{ij}=0$.

We see that all feature matrices have a similar shape. The largest feature is $D_{10,1}$ in the bottom left corner, as expected, because it is the average degree increment of the top-10% degree vertices obtained from the last, by far the largest (due to the exponential growth of the network in calendar time), time-cohort. The values of the other features gradually reduce with their distance to $D_{10,1}$. The top-right corner is always empty, conform the old-get-richer phenomenon: low-degree vertices did not get any link early on. Surprisingly, this is true even for the Uniform model and for the models with aging. While all feature matrices have a similar shape, it turns out that the classifier discriminates the models by the differences between the numbers in the cells. We visualize this in Figure 4 as follows. For each feature $D_{ij}$ we compute its average over all synthetic networks, $\bar{D}_{ij}$. Then for each model, we plot the relative difference $\delta _{ij}=(D_{ij}-\bar{D}_{ij})/\bar{D}_{ij}$. Note that for fixed *i*, *j*, the sum of $\delta _{ij}$ over all models equals zero because $\bar{D}_{ij}$ is the average over the nine models. However, the elements of the $\delta$ matrix for a given model are not comparable because they are normalized by different values of $\bar{D}_{ij}$.

The matrices in Figure 4 are strikingly different. This explains the success of the classifier. Moreover, Figure 4 yields several interesting insights. First, note that the largest negative value of $\delta _{ij}$ is $-1$, when $D_{ij}=0$. In particular, in the Uniform model, and all models with *fitness* but without *preferential attachment*, the last column has only $-1$’s, so at least 10% vertices in these models have in-degree zero. As expected, the models with *aging* smooth out the degree increments. Indeed, we see that $\delta$ has values closer to zero, in other words, if $D_{ij}$’s in the models with aging are relatively close to $\bar{D}_{ij}$. Next, we now see that in Figure 4, the ‘old-get-richer’ effect is most prominent in the pure *preferential attachment* model, P. Indeed, low-quantile vertices are the ones that are born late in the process because this is when they start gaining positive degree increments.

**Classification results with static and dynamic features.** With static and dynamic features together, the accuracy increases to $98.40\pm 0.3$%. It is on average higher than with the dynamic features alone, but the error margins do overlap. The confusion matrix is shown in Figure 2c. We conclude that adding static features does not significantly improve the accuracy.

### Classification applied to citation networks

We use citation network data from the Web of Science. Our dataset contains publications between 1980 and 2015. Citations are included only to the papers contained in the dataset. In order to obtain somewhat homogeneous real-life networks, we use citation networks separately for different scientific fields. See the brief summary of the data in Table 1.

**Table 1:** The summary of the Web of Science citation networks for several scientific fields.

Acronym	Field	Original size	Size of connected component
AP	Astrophysics	477113	451730
BT	Biotechnology	537867	421643
PS	Probability and statistics	185167	157500
GE	Geology	56692	46672
NP	Nuclear physics	223321	198162
OC	Organic chemistry	567146	535945
OP	Optics	501817	416893
SO	Sociology	222416	91736

In the experiments, only the connected component was used.

Section A.1 in the Supplementary Material contains detailed discussion of typical empirical properties of citation networks reported in the literature.

The classifier takes the dynamic citation network as input and returns the probability distribution over the nine categories. Table 2 shows the most likely category for each research field using static features, dynamic features and the combination thereof. For the complete distribution over classes, see Tables A.2–A.5 in the Supplementary Material.

With static features only, citation networks are classified ${\hbox {F}_{\textsc {exp}}}{\hbox {A}}$ or AP. This is surprising because neither of these growth mechanisms gives rise to power-law in-degrees. With the size-cohorts dynamic features or the combination of the static features and the size-cohorts dynamic features, most citation networks are classified as ${\hbox {F}_{\textsc {exp}}}{\hbox {AP}}$, the model that has been suggested in the literature^24^. Interestingly, precisely *this* combination of mechanisms makes the in-degrees have dynamic power-laws, where the exponent decreases over time to some limit^14^, as observed in the data (see Figure A.3a in the Supplementary Material). SO is clearly different, it is the only citation network never being classified as ${\hbox {F}_{\textsc {exp}}}{\hbox {AP}}$. Furthermore, it is classified as P by dynamic features with size-cohorts, which is the only classification that does not involve aging. Notably, the results with time-cohorts dynamic features differ from those with size-cohorts dynamic features; the citation networks are often classified as AP and sometimes ${\hbox {F}_{\textsc {exp}}}{\hbox {A}}$. Altogether, with the best classifiers, we find it likely that aging is part of the real-world mechanisms, but whether this is combined with preferential attachment, exponential fitness, or both, is inconclusive. It is also clear that ${\hbox {F}_{\textsc {exp}}}$ appears statistically most likely out of the considered fitness options.

**Table 2:** Classification results on WoS citation networks.

Static Features		Yes	No	Yes	No	Yes
Dynamic Features		No	Size-cohorts	Size-cohorts	Time-cohorts	Time-cohorts
Accuracy on synthetic data		92.81%	97.33%	97.62%	98.06%	98.40%
AP	Astrophysics	${\hbox {F}_{\textsc {exp}}}{\hbox {A}}$	${\hbox {F}_{\textsc {exp}}}{\hbox {AP}}$	${\hbox {F}_{\textsc {exp}}}{\hbox {AP}}$	AP	${\hbox {F}_{\textsc {exp}}}{\hbox {A}}$
BT	Biotechnology	AP	${\hbox {F}_{\textsc {exp}}}{\hbox {AP}}$	${\hbox {F}_{\textsc {exp}}}{\hbox {AP}}$	AP	${\hbox {F}_{\textsc {exp}}}{\hbox {AP}}$
GE	Geology	AP	${\hbox {F}_{\textsc {exp}}}{\hbox {AP}}$	AP	AP	AP
NP	Nuclear Physics	${\hbox {F}_{\textsc {exp}}}{\hbox {A}}$	${\hbox {F}_{\textsc {exp}}}{\hbox {AP}}$	${\hbox {F}_{\textsc {exp}}}{\hbox {AP}}$	AP	${\hbox {F}_{\textsc {exp}}}{\hbox {A}}$
OC	Organic Chemistry	${\hbox {F}_{\textsc {exp}}}{\hbox {A}}$	${\hbox {F}_{\textsc {exp}}}{\hbox {AP}}$	${\hbox {F}_{\textsc {exp}}}{\hbox {AP}}$	AP	${\hbox {F}_{\textsc {exp}}}{\hbox {A}}$
OP	Optics	AP	${\hbox {F}_{\textsc {exp}}}{\hbox {AP}}$	${\hbox {F}_{\textsc {exp}}}{\hbox {AP}}$	AP	${\hbox {F}_{\textsc {exp}}}{\hbox {AP}}$
PS	Probability and Statistics	AP	${\hbox {F}_{\textsc {exp}}}{\hbox {AP}}$	${\hbox {F}_{\textsc {exp}}}{\hbox {AP}}$	AP	${\hbox {F}_{\textsc {exp}}}{\hbox {AP}}$
SO	Sociology	AP	P	AP	AP	AP

## Discussion

**Excellent classification of dynamic network models.** The classification of the synthetic data based on networks dynamics has excellent accuracy higher than 98%, greatly superseding the accuracy with state-of-the-art static features (92.8%). The accuracy may improve only slightly when adding static features to the dynamic ones. Our dynamic features are interpretable in that we identify clear differences between distinct models. This is a highly desirable property for machine learned model selection.

**Application to citation networks.** Using dynamic features, ${\hbox {F}_{\textsc {exp}}}{\hbox {AP}}$ is often the most likely classification for citation networks. SO is an outlier in that it is never classified ${\hbox {F}_{\textsc {exp}}}{\hbox {AP}}$. However, in some cases, other models are also quite plausible in terms of the likelihood of their classification. For instance, in Table A.6 in the Supplementary Material we see that the classifier (with both static features and dynamic features with size-cohorts) estimates the likelihood of uniform attachment (U) for OC as 47.484%, which is only slightly smaller than the likelihood 50.6094% of ${\hbox {F}_{\textsc {exp}}}{\hbox {AP}}$. This demonstrates that the most likely classification of real-life networks is not always robust, the entire distribution over the nine models is more informative. Furthermore, in practice, we recommend to use both dynamic features designs – with size-cohorts and time-cohorts – for more reliable conclusions. In particular, in our application to citation networks, all feature choices have clear preferences for just a few classes (${\hbox {F}_{\textsc {exp}}}{\hbox {AP}}$, AP, sometimes ${\hbox {F}_{\textsc {exp}}}{\hbox {A}}$ and P). At the same time, many other classes are clearly ruled out, e.g., the quite plausible ${\hbox {F}_{\textsc {pl}}}{\hbox {A}}$ and most models without aging, see Tables A.2–A.6).

In search for a simple explanation why different features result in a different classification on real-life networks, we have performed many additional experiments, for instance, computing distances between the synthetic and real-life feature vectors. These attempts however were not successful in the sense that simple measures like distances did not explain the classification. As an example, and for completeness, we report the results of our best attempt, the two-dimensional UMAP (Uniform Manifold Approximation and Projection) embeddings of the features, see Sect. A.10 of the Supplementary Material. We see that the two-dimensional embeddings of different synthetic classes have significant overlap. This makes classification of citation networks with UMAP highly unreliable, and exemplifies the complexity of learning the right model, even for *synthetic* networks. Similarly, in our experiments with other known methods, the complex structure of our features could not be captured in spaces of much lower dimensions.

**Power laws in networks.** Interestingly, by^14^, the ${\hbox {F}_{\textsc {exp}}}{\hbox {AP}}$ model, that is often selected by our classifier for citation networks, has dynamical power-law in-degrees with exponent decreasing over time. This is a new argument in favor of using power laws as a mathematical model for degree distributions. There is a lively debate about whether power-law in-degrees are rare or ubiquitous (see^40–42^ and the references therein). One of the problems with statistical evaluation of power laws is that, fundamentally, a power law holds only in the infinite network size limit ^41^. Model selection offers an alternative, albeit arguably qualitative, approach to infer the presence of power laws: first, identify the model based on a finite network; then analyze this model when the network size grows to infinity.

**A cautionary tale on ML techniques for model selection.** Our machine learning methods work really well to distinguish between synthetic networks having different growth mechanisms. Moreover, these methods are general in that they identify the combination of mechanisms regardless the exact value of parameters. However, applying these methods to real-world networks with the aim to distinguish network growth mechanisms may lead to non-robust results because models are never perfect representations of the real-world networks, and machine-learning methods pick up differences easily. We believe that the true reason for this sensitivity is that in reality, citation networks are not accurately described at a detailed level by any of the models considered here. Therefore, depending on the features, the model selection yields the result that is *most consistent* with the chosen features. Hence, the best modeling choice depends on which features we want to capture. This calls for systematic approaches to feature design depending on a specific research question and application, as well as validation methods of machine learned model selection.

**Further research.** It would be of interest to study *other* possible generative models. Particularly for citation networks, models with *copying* ^43^ or high probability of triangles ^19^ might be appropriate. This will account for the situation when authors cite both a paper and some references from that paper. One may also want to include geometry and make closer vertices more likely to connect ^44^ mimicking research topics and communities. It would be highly interesting to find a set of mechanisms that will be consistently selected by different classifiers for all citation networks, if such a combination of mechanisms exists. Furthermore, we may consider networks where links between fixed vertices change over time as many related real-world networks, such as social networks and the World Wide Web.

Our dynamic features are computationally light, this enables further investigation of machine learning methods. For instance, one could include varying network sizes, explore other approaches to feature importance (e.g. Shapley values), and use regression to fit model parameters for real-life networks. Moreover, our features have explicit analytical expressions, thus we may hope to analytically derive the accuracy of machine learning methods and identify most informative features using the theory of continuous-time branching processes.

In terms of model selection, our cautionary tale requires further investigation. Our results show that model selection is sensitive to the choice of features. This calls for new methods of systematic feature design that is suitable for the problem at hand. New experiments can be done to go further in details in choosing the best classifier (e.g. training binary classifiers which predict the presence or absence of a dynamic network mechanism such as aging, broader parameter range for simulations, larger tuning hyperspace).

## Methods

### Collapsed CTBP models for dynamic networks

**Modeling growth mechanisms with CTBPs.** Our models build on a continuous-time branching process (CTBP) representation of preferential attachment models. The equivalence of these processes was established, for instance, in  ^45^, Theorem 3.3 and^46^, Theorem 2.1. Furthermore, collapsed CTBPs represent the local weak limit of directed PAMs as established in^47^, Theorem 1 and^48^, Proposition 6.10, see also a unified treatment of this result in^10^, Section 4.2. The CTBP’s are random growing trees. By defining the rates, at which vertices of a CTBP produce their offspring, one can model desired mechanisms of network’s growth. In this work, the rates explicitly depend on the *fitness* of the vertex, its *age*, and its *degree* (preferential attachment). Fix a vertex *u* of CTBP, and let $t_u$ denote its birth time. Then, the rate at which vertex *u* produces offspring at time *t* is given by

1 $$\begin{aligned} {\eta _u h(t-t_u) f(\text{ in-degree}_u(t)),\quad t>t_u,} \end{aligned}$$

where $h:[0,\infty )\rightarrow [0,\infty )$ denotes the aging function, $\eta _u$ the fitness of vertex *u*, *f* the preferential attachment function, and $\text{ in-degree}_u(t)$ is the in-degree, that is, number of offspring of vertex *u* at time *t*. This gives a flexible class of CTBPs models for aging, fitness, and preferential attachment, moreover, we can switch off any of these mechanisms by taking *h*, $\eta _u$ and/or *f* equal to 1. Motivated by empirical properties of citation networks (see Sect. A.1 in the Supplementary Material), we make the following choices in (1).

The *preferential attachment function* *f*, when present, is *affine*, a key example being the linear preferential attachment function used in the Barabási–Albert model^11^. We take $f(k)=ak+b$, $a,b>0$.

The *fitness variables* $(\eta _v)_{v\in V(T)}$ are positive i.i.d. random variables. In the FP models, the distribution of $\eta _v$’s must have finite support, since otherwise condensation occurs that is absent in citation networks ^38,49^. Therefore, we assume the uniform distribution on the interval [*c*, *d*]. In FAP models, in order to obtain sufficient variability in the network’s degree distribution, $\eta _v$’s must have unbounded support, with at least an exponential tail ^14^, therefore we assume the exponential distribution with parameter $\lambda$. Finally, in F and FA models, we consider $\eta _v$’s to have either the exponential distribution or a power law, that is pure Pareto with density $(\tau -1)x_{\textrm{min}}^{\tau -1}x^{-\tau }$, $x>x_{\textrm{min}}$.

We let the *aging function* *h*, when present, be the density of a log-normal distribution conform to the log-normal aging as in Figure A.13,

$$\begin{aligned} h(t)=\frac{1}{x\sigma \sqrt{2\pi }}\,e^{-\frac{(\ln (t)-\mu )^2}{2\sigma ^2}}, \qquad t>0. \end{aligned}$$

**From trees to graphs with collapsed CTBP’s.** A CTBP produces a tree, which we convert to a graph *G*(*t*) with out-degrees greater than 1, using the following collapsing procedure.

First, we generate random out-degrees $M_1,M_2,\ldots$ as independent copies of a random variable *M* sampled from the empirical distribution of the number of references in a paper in the WoS OC network, with $\mathbb {E}[M] \approx 10.29$. The OC network was chosen because it is sufficiently representative for the out-degree distributions in the other citation networks as well. See Sect. A.2 in the Supplementary Material for details.

Next, we start generating the CTBP, simultaneously assigning its vertices to *batches* of sizes $M_1,M_2,\ldots$ in the order of their arrival. At time *t*, graph *G*(*t*) is produced by collapsing every batch into one vertex *v* of *G*(*t*). When generating the CTBP, all vertices in the batch of *v* receive the same fitness $\eta _v$ and the same birth time $t_v$, equal to the birth time of the *oldest* CTBP vertex in the batch. Furthermore, we make the total rate, at which a collapsed vertex *v* produces offspring, independent of its out-degree $M_v$, by distributing the original rate (1) equally among the $M_v$ collapsed vertices. Summarising, in our collapsed model, every vertex *u* of the CTBP, that is to be collapsed into vertex *v* of *G*(*t*), produces offspring at rate

2 $$\begin{aligned} {\frac{1}{M_v}\eta _v h(t-t_v) f(\text{ in-degree}_u(t)),\quad t>t_v.} \end{aligned}$$

In application to citation networks, each collapsed vertex *v* models one paper; this paper cites $M_v$ other papers; the publication date is the birth time of the oldest collapsed vertex; the citations to this paper are the offspring produced by the $M_v$ collapsed vertices together.

**Parameters range of the collapsed CTBP.** Table 3 summarises the generative models, on which we have trained our machine-learning methods. The last column (**‘Power law’**) states which models have power-law in-degrees, based on the literature and Proposition 1 in Sect. A.5 of the Supplementary Material. Column **‘Range’** displays the parameter ranges that we used for different models. These ranges are derived from the following considerations. There are only three possible scenarios of the CTBP’s growth: 1) CTBP is *subcritical*, it dies out after producing a random finite tree, 2) CTBP is *supercritical*, it grows exponentially in time with positive probability, or 3) CTBP is *explosive*, it produces an infinite tree in a finite time. Since real-life citation networks grow exponentially in time (as in Figure A.1a in the Supplementary Material), we choose the range of parameters $a,b,c,d,\lambda , x_{\textrm{min}},\tau , \mu ,\sigma$ inside the region where the CTBP with the original rate (1) is supercritical. We derive these conditions in Sect. A.3, and discuss the supercriticality of the collapsed process in Sect. A.4 of the Supplementary Material. In the models with aging, a supercritical branching process dies out with positive probability. In the experiments, for each parameter combination, we remove networks that die out, and keep the first network that achieves a size of 20, 000 within at most 1000 attempts. For example, Figure A.5 in the Supplementary Material shows the number of ${\hbox {F}_{\textsc {pl}}}$A networks that died out for different values of $\tau$. For simplicity of interpretation, we choose the parameter range so that the average rate of producing offspring is similar across different models. Finally, we choose the models such that the CTBP cannot be explosive. Thus, we exclude FP models with exponential or power law fitness, as such models do explode in finite time^38^.

**Table 3:** Dynamic network models with parameters. The parameter range is chosen so that the CTBP is supercritical, i.e., it grows exponentially in time (see Sect. A.3 for details).

Code	Name	Growth mechanism	Parameters	Range	Power-law
0	U	Uniform attachment	None	–	No ^50^, Exercise 8.17
1	P	Affine PA	*a*, *b*	$a \in (1, 4), b \in (1, 4)$	Yes ^50^, Theorem 8.3
2	$\textsc {F}_{\textsc {pl}}$	Power-law fitness	$(x_{\textrm{min}}, \tau )$	$x_{\textrm{min}} \in (0.5, 1), \tau \in (2, 4)$	Yes (Proposition A.1)
3	$\textsc {F}_\textsc {exp}$	Exponential fitness	$\lambda$	$\lambda \in (0.1, 3)$	No (Proposition A.1)
4	$\textsc {F}_\textsc {pl}$A	Power-law fitness lognormal aging	$(x_{\textrm{min}}, \tau )$ $(\mu , \sigma )$	$x_{\textrm{min}} \in (0.5, 1), \tau \in (2, 2.7)$ $\mu \in (0.1, 3), \sigma = 1$	Yes (Proposition A.1)
5	$\textsc {F}_\textsc {exp}$A	Exponential fitness Lognormal Aging	$\lambda$ $(\mu , \sigma )$	$\lambda \in (0.1, 1)$ $\mu \in (0.1, 3), \sigma = 1$	No (Proposition A.1)
6	$\textsc {F}_\textsc {unif}$P	Affine PA uniform fitness	(*a*, *b*) (*c*, *d*)	$a \in (1, 4), b \in (1, 4)$ $c \in (0.1, 1), d \in (1, 5)$	Yes^38^, Theorem 3
7	AP	Affine PA lognormal aging	(*a*, *b*) $(\mu , \sigma )$	$a \in (3.3, 7), b \in (1, 4)$ $\mu \in (0.1, 3), \sigma = 1$	No^14^, Section 5.1
8	$\textsc {F}_\textsc {exp}$AP	Affine PA Exponential fitness Lognormal aging	(*a*, *b*) $\lambda$ $(\mu , \sigma )$	$a \in (1, 4), b \in (1, 4)$ $\lambda \in (0.1, a + \frac{b}{{\mathbb {E}}\left[ M\right] })$ $\mu \in (0.1, 3), \sigma = 1$	Yes^14^, Section 5.2

### Machine learning methodology

**Static features.** The 36 static features are selected from recent literature. We compute static features on the *undirected* version of our networks because most of these features are designed for undirected graphs. Two static features are global characteristics of the graph: the assortativity coefficient^15,23,39^ is the Pearson’s correlation coefficient between degrees of two vertices connected by an edge; the global clustering coefficient (or, transitivity)^15,16,39^ is the fraction of triangles among connected triplets of vertices. Other features describe the distributions of numerical *values* of individual vertices. For each value we compute the minimum, the maximum, the mean, the standard deviation, and 3 to 5 quantiles that proved descriptive for different networks. Two values are vertex centralities: in-degrees^15,16,21,23^ (quantiles 0.125, .25, .5, .75, .875), and coreness^21,23^ (quantiles .25, .5, .75). The coreness of a vertex equals the largest number *k* such that the vertex is in the *k*-core – the maximal induced subgraph with all vertices having degree *k* or higher. Another type of values describes the vertex neighborhoods. Of these, we use the number of triangles that contain the vertex^16,23^ (quantiles .80, .90, .95, .97, .99, which have sufficient variance to be used as features), and the local clustering coefficient^21,23^ (quantiles .5, .6, .7, .8, .9). The latter is the fraction of pairs of neighbors of the vertex that are connected to each other. Our features do not need to be independent of the network size^39^, because all synthetic networks in our training set have the same size.

We do not include edge density^21^, because our networks are of comparable density defined by the average in/out-degree ${\mathbb {E}}\left[ M\right]$. We do not use PageRank^16,21^ and Katz index^21^, because of their similarity to in-degree in our models. Diameter^21^, closeness and betweenness centrality^21^ and motif counts^16,39^ are excluded because their infeasible computational costs on many networks of size 20,000. Finally, our synthetic networks are not expected to contain communities, thus we omit the number of communities^16^.

**Novel dynamic features.** A core contribution of this paper is the *dynamic feature design* that directly summarizes the *evolution* of the dynamic graph $G(t)=(V(t), E(t))$, $t\in (0,T]$. Our dynamic features form a Dynamic Feature Matrix (DFM) *D* of size $s \times r$, defined as follows. The *columns* $j\in [r]$ of the matrix *D* correspond to groups of vertices $V_j$ between $(r-j)/r$-th and $(r-j+1)/r$-th quantiles of the empirical degree distribution at time *T*. Formally,

$$\begin{aligned} V_{j} = \Big \{&v \in V(T) :\frac{r-j}{r} < \frac{1}{|V(T)|} \sum _{w \in V(T)}\mathbbm {1}\{\deg _{w}(T)\le \deg _{v}(T)\} \le \frac{r-j+1}{r} \Big \}, \; j \in [r]. \end{aligned}$$

For instance, $V_1$ contains the vertices whose degree at time *T* is in the highest (100/*r*)% of *V*(*T*).

The *rows* $i\in [s]$ of the matrix *D* stand for cohorts of vertices in the order of their arrival. More specifically, we define time instants $0=\tau _0<\tau _1<\cdots <\tau _s=T$, and let cohort *i* consist of those vertices that have arrived in the time interval $(\tau _{i-1},\tau _i]$. To define the $\tau _i$’s, we consider *size*-cohorts and *time*-cohorts. Size-cohorts all contain the same number of vertices, and $\tau _i$ is the time when the $\lfloor |V(T)| i/s \rfloor$-th vertex arrives. Time-cohorts correspond to the calendar time, they consist of vertices that have arrived in equal time intervals, and $\tau _i = Ti/s$. Note that in the CTBP, the number of vertices in time-cohort *i* increases exponentially in *i*, so the highest-degree vertices in $V_1$ attract many edges from the last, the largest, time-cohort.

The *entries* *ij* of the matrix *D* are the normalized average number of edges received by vertices in $V_j$ from cohort *i*:

3 $$\begin{aligned} D_{ij} = C\Delta _{ij}, \quad i\in [s], j\in [r], \end{aligned}$$

where

4 $$\begin{aligned} \Delta _{ij}= \frac{1}{|V_j|}\sum _{v \in V_j}(\deg _{v}(\tau _{i}) - \deg _{v}(\tau _{i-1})), \end{aligned}$$

and *C* is the normalization constant so that $\sum _{i=1}^s\sum _{j=1}^rD_{ij}=1$. This normalization is a common practice to prevent overfitting. In our case, $\Delta _{ij}$ increases with the graph size, and, by construction, decreases in *s* and *r*. The normalization ensures that our learned classes are robust with respect to the choice of |*V*(*T*)|, *s* and *r*. Intuitively, the dynamic features are meaningful for model selection because different models prescribe different likelihood of vertices to attract new edges. This is exactly what we are able to track with dynamic features.

**Choice of classifier** is explained in Sect. A.7 of the Supplementary Material.

### Supplementary Information

Supplementary Information.

## Data Availability

The dataset with synthetic networks and their static and dynamic features: DOI 10.4121/0b40e329-7b33-4b8f-a289-ab6d1893b437 available at https://data.4tu.nl/datasets/0b40e329-7b33-4b8f-a289-ab6d1893b437.

## Code availability

Code repository for network simulation and feature extraction: https://gitfront.io/r/user-6239985/R9WcT8Msr46T/DynamicNetworkSimulation/.

## References

[1] Dial R, Glover DF, Karney D, Klingman D (1979). A computational analysis of alternative algorithms and labeling techniques for finding shortest path trees. Networks.

[2] Madduri, K., Bader, D. A., Berry, J. W. & Crobak, J. R. An experimental study of a parallel shortest path algorithm for solving large-scale graph instances. In *Proc. Ninth Workshop on Algorithm Engineering and Experiments (ALENEX)* 23–35. 10.1137/1.9781611972870.3 (2007).

[3] Niewenhuis, D. & Varbanescu, A.-L. “Efficient trimming for strongly connected components calculation”. In Proc. *19th ACM International Conference on Computing Frontiers*, pp. 131–140 (2022).

[4] van der Hofstad R, Komjáthy J (2017). When is a scale-free graph ultra-small?. J. Stat. Phys..

[5] Pastor-Satorras R, Vespignani A (2001). Epidemic spreading in scale-free networks. Phys. Rev. Lett..

[6] Chatterjee S, Durrett R (2009). Contact processes on random graphs with power law degree distribution have critical value 0. Ann. Probab..

[7] Albert R, Jeong H, Barabási AL (2000). Error and attack tolerance of complex networks. Nature.

[8] Doyle JC, Alderson DL, Li L (2005). The “robust yet fragile” nature of the internet. Proc. Nat. Acad. Sci..

[9] Chen N, Litvak N, Olvera-Cravioto M (2017). Generalized PageRank on directed configuration networks. Random Struct. Algorithms.

[10] Banerjee S, Olvera-Cravioto M (2022). PageRank asymptotics on directed preferential attachment networks. Ann. Appl. Probab..

[11] Barabási AL, Albert R (1999). Emergence of scaling in random networks. Science.

[12] Mahadevan P, Krioukov D, Fall K, Vahdat A (2006). Systematic topology analysis and generation using degree correlations. ACM SIGCOMM Comput. Commun. Rev..

[13] Krioukov D, Papadopoulos F, Kitsak M, Vahdat A, Boguná M (2010). Hyperbolic geometry of complex networks. Phys. Rev. E.

[14] Garavaglia A, van der Hofstad R, Woeginger G (2017). The dynamics of power laws: Fitness and aging in preferential attachment trees. J. Stat. Phys..

[15] Attar N, Aliakbary S (2017). Classification of complex networks based on similarity of topological network features. Chaos Interdiscip. J. Nonlinear Sci..

[16] Langendorf RE, Burgess MG (2021). Empirically classifying network mechanisms. Scie. Rep..

[17] Gao, F. & van der Vaart, A. “Statistical inference in parametric preferential attachment trees,” *arXiv preprint* arXiv:2111.00832, (2021).

[18] Gómez V, Kappen HJ, Litvak N, Kaltenbrunner A (2013). A likelihood-based framework for the analysis of discussion threads. World Wide Web.

[19] Overgoor, J., Benson, A. & Ugander, J. “Choosing to grow a graph: Modeling network formation as discrete choice”. In *The World Wide Web Conference*, pp. 1409–1420, (2019).

[20] Dehmamy, N., Barabási, A.-L. & Yu, R. Understanding the representation power of graph neural networks in learning graph topology. *Adv. Neural Inf. Process. Syst.* **32** (Curran Associates, Inc., 2019).

[21] Bläsius, T., Friedrich, T., Katzmann, M., Krohmer, A. & Striebel, J. Towards a Systematic Evaluation of Generative Network Models. *Lecture Notes in Computer Science (Including Subseries Lecture Notes in Artificial Intelligence and Lecture Notes in Bioinformatics)* **1083 LNCS6**, 99–114 (2018).

[22] Canning JP, Ingram EE, Nowak-Wolff S (2018). Predicting Graph Categories from Structural Properties.

[23] Rossi RA, Ahmed NK (2019). Complex networks are structurally distinguishable by domain. Soc. Netw. Anal. Min..

[24] Wang D, Song C, Barabási AL (2013). Quantifying long-term scientific impact. Science.

[25] Garavaglia, A. “Preferential Attachment Models for Dynamic Networks”. Ph.D. dissertation, Technische Universiteit Eindhoven, Eindhoven, (2019).

[26] Yasui Y, Nakano J (2022). A stochastic generative model for citation networks among academic papers. Plos One.

[27] Chang LL-H, Phoa FKH, Nakano J (2021). A generative model of article citation networks of a subject from a large-scale citation database. Scientometrics.

[28] Zhou B, Holme P, Gong Z, Zhan C, Huang Y, Lu X, Meng X (2023). The nature and nurture of network evolution. Nat. Commun..

[29] Bonner, S., Brennan, J., Theodoropoulos, G., Kureshi, I. & McGough, A. S. “Deep topology classification: A new approach for massive graph classification”. In Proc. *2016 IEEE International Conference on Big Data, Big Data 2016*, pp. 3290–3297, (2016).

[30] Hegde, K., Magdon-Ismail, M., Ramanathan, R. & Thapa, B. “Network signatures from image representation of adjacency matrices: Deep/transfer learning for subgraph classification,” *arXiv preprint* arXiv:1804.06275, (2018).

[31] Wang D, Song C, Barabási A-L (2013). Quantifying Long-term scientific impact. Science.

[32] Rossi, R. A. & Ahmed, N. K. “The network data repository with interactive graph analytics and visualization”. In *AAAI*, (2015). [Online]. Available: https://networkrepository.com.

[33] Wang M, Yu G, Yu D (2009). Effect of the age of papers on the preferential attachment in citation networks. Phys. Stat. Mech. Appl..

[34] Price D J d S (1965). Networks of scientific papers. Science.

[35] Wang M, Yu G, Yu D (2008). Measuring the preferential attachment mechanism in citation networks. Phys. Stat. Mech. Appl..

[36] Jagers, P. *Branching processes with biological applications*. London: Wiley-Interscience [John Wiley & Sons], pp. xiii+268, Wiley Series in Probability and Mathematical Statistics—Applied Probability and Statistics, (1975) isbn: 0-471-43652-6.

[37] Jagers, P. & Nerman, O. “The asymptotic composition of supercritical multi-type branching populations”. In *S é minaire de Probabilit é s, XXX*, ser. Lecture Notes in Math. Vol. **1626**, Berlin: Springer, pp. 40–54 (1996).

[38] Borgs, C., Chayes, J., Daskalakis, C. & Roch, S. “First to market is not everything: An analysis of preferential attachment with fitness”. In Proc. *Thirty-Ninth Annual ACM Symposium on Theory of Computing*, pp. 135–144 (2007).

[39] Ikehara, K. & Clauset, A. “Characterizing the structural diversity of complex networks across domains,” (2017). [Online]. Available: https://arxiv.org/abs/1710.11304v1.

[40] Broido A, Clauset A (2019). Scale-free networks are rare. Nat. Commun..

[41] Voitalov I, van der Hoorn P, van der Hofstad R, Krioukov D (2019). Scale-free networks well done. Phys. Rev. Res..

[42] Holme P (2019). Rare and everywhere: Perspectives on scale-free networks. Nat. Commun..

[43] Kumar, R., Raghavan, P., Rajagopalan, S., Sivakumar, D., Tomkins, A. & Upfal, E.“Stochastic models for the web graph”. In Proc. *41st Annual Symposium on Foundations of Computer Science*, IEEE, pp. 57–65 (2000).

[44] Flaxman AD, Frieze AM, Vera J (2006). A geometric preferential attachment model of networks. Internet Math..

[45] Athreya KB (2007). Preferential attachment random graphs with general weight function. Internet Math..

[46] Athreya KB, Ghosh AP, Sethuraman S (2008). Growth of preferential attachment random graphs via continuous-time branching processes. Proc. Indian Acad. Sci. Math. Sci.

[47] Rudas A, Tóth B, Valkó B (2007). Random trees and general branching processes. Random Struct. Algorithms.

[48] Garavaglia A, Van der Hofstad R, Litvak N (2020). Local weak convergence for PageRank. Ann. Appl. Probab..

[49] Bianconi G, Barabási A-L (2001). Bose–Einstein condensation in complex networks. Phys. Rev. Lett..

[50] Van der Hofstad R (2017). Random Graphs and Complex Networks.

