# Supplementary material for: Learning the mechanisms of network growth
Source: Sci Rep. 2024 May 24;14:11866. doi: 10.1038/s41598-024-61940-4 (PMC11126688; doi:10.1038/s41598-024-61940-4)
Supplement: Supplementary file 1 — Supplementary Information. [file 41598_2024_61940_MOESM1_ESM.pdf]

# Supplementary Material: Learning the mechanisms of network growth

Lourens Touwen[a,1], Doina Bucur[b,2], Remco van der Hofstad[a,3], Alessandro Garavaglia[a,4], and Nelly Litvak[a,5,*]

[a]Department of Mathematics and Computer Science, Eindhoven University of Technology, Groene Loper 3, 5612 AE Eindhoven, The Netherlands
[b]Faculty of Electrical Engineering, Mathematics and Computer Science, University of Twente, Drienerlolaan 5, 7522 NB, Enschede, The Netherlands
*Email address*: [1]touwenlourens@gmail.com, [2]d.bucur@utwente.nl, [3]rhofstad@win.tue.nl, [4]ale.garavaglia@gmail.com, [5]n.v.litvak@tue.nl, * corresponding author

May 21, 2024

# A Supplementary Material

## A.1 Empirical properties of the evolution of citation networks

In citation networks, vertices are scientific publications, and directed edges are citations from one publication to another. The structure of citation networks has received tremendous attention, ever since the groundbreaking work of De Solla Price [Pri65; Pri86]. Recent studies of citation networks include [WSB13; WYY08; WMH14; GHW17]. Here we discuss some of the properties of citation networks that form the basis of the network models we consider in this paper.

**Citation networks grow exponentially in calendar time.** We illustrate this with examples from the fields: Biotechnology (BT), Probability and statistics (PS). These examples were generated by us for the new book [Hof23], we include them here to make the current paper self-contained, and refer the reader to [Hof23] for more details. Figure A.1 displays the exponential growth in citation networks in the fields BT and PS.

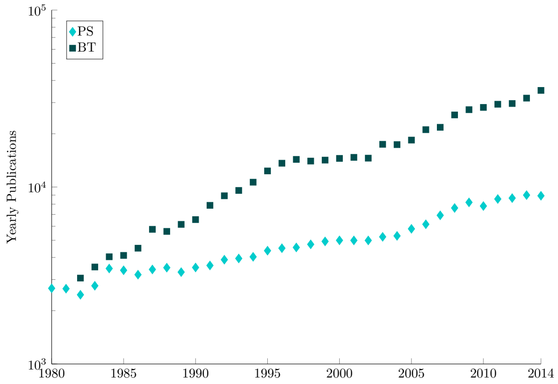


Figure A.1: Number of publications per year in the fields (logarithmic vertical axis).

**Citation networks have highly variable degrees.** Figure A.2 shows the citation evolution of 20 random papers from 1980. While this is an anecdotal example, we do see that some papers keep on attracting citations, while others stop being cited quite quickly. Again, this is a sign of the highly-variable degree evolution. Motivated by the obviously high variability of citation counts, a vast body of literature starting with Price [Pri65] has suggested power-law in-degree distributions in citation networks. The question whether power-laws in networks are 'rare' or 'omnipresent' has caused considerable scientific debate [Art+20; BC19; Hol19; Voi+19]. For our data, the log-log plots in Figure A.1(b) suggests that the in-degree distribution could be a power-law, even though the tails might also be slightly thinner. As time proceeds, important papers attract more citations making power laws more visible. Figure A.3(a) shows the *dynamics* of in-degree distribution in BT from a sample of papers published in 1984. We see a strong time dependence, in that the degree distribution of a cohort of papers from a given time period (in this case the year 1984) becomes more and more heavy-tailed.

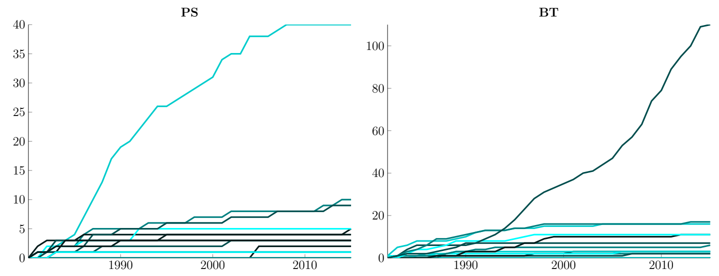


Figure A.2: Time evolution for citations of 20 randomly chosen papers from 1980 for PS, and from 1982 for BT.

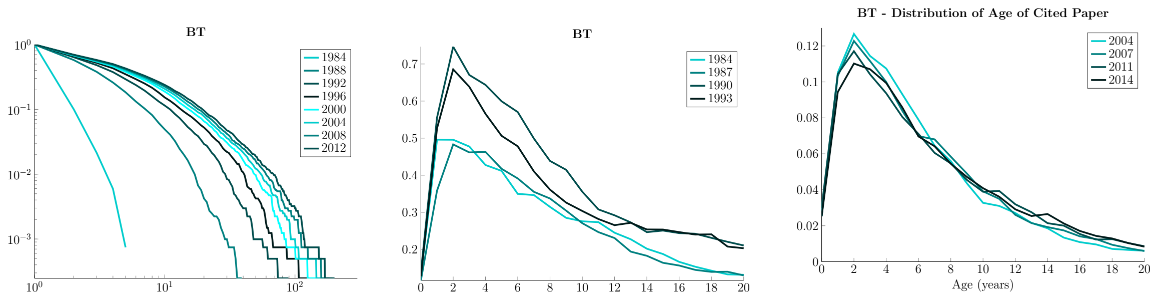


(a) (b) (c)

Figure A.3: Empirical properties of BT citation network [Hof23]. (a). Degree distribution for papers from 1984 over time. Each log-log plot shows the citation distribution in a given later year. As time progresses, the number of citations naturally increases in a way that the tail becomes significantly heavier, stabilizing after a long time. This is modeled with dynamic power laws: the number of citations follows a power law, and its exponent is decreasing with time. (b). Average citation increment over a 20-years time window for papers published in different years show aging effect. (c). Distribution of the age of cited papers for different citing years. We have used a 20-year time window in order to compare different citing years. The shape of the distribution for different citing years in each field is very similar and resembles a log-normal density.

**Citation increments depend on the age of a paper.** In citation networks, the majority of papers stop receiving citations after some time, while few others keep being cited for longer times (see e.g., Figure A.2 for the citation patterns of a few papers). Typically, the average increment of citations increases first, and then decreases over time (see Figure A.3(b). This is a manifestation of *aging*. Moreover, in Figure A.3(c), we plot, as an example, the distribution of the age of cited papers, by papers published in a given year. The plot suggests a log-normal distribution of the age of a cited paper. Notably, the shape of this log-normal distribution is rather stable over time, and is similar for different scientific fields.

## A.2 Empirical out-degree distribution used in the synthetic networks

Recall that we denote by $M$ a random variable that represents the out-degree of a vertex in synthetic networks. To model this random variable, we use the empirical distribution of the number of references in a paper in the Web of Science OC network. The OC network was chosen as its out-degree distribution is representative for the other out-degree distributions of the other citation networks. Importantly, $M$ counts references only to the papers in the dataset, so it is smaller than the total number of references in a paper. Figure A.4 shows the empirical mass function and cumulative distribution function of $M$. Table A.1 provides the statistical summaries of this empirical distribution. The low mode (equal 1) is due to the fact that we count only references within the dataset.

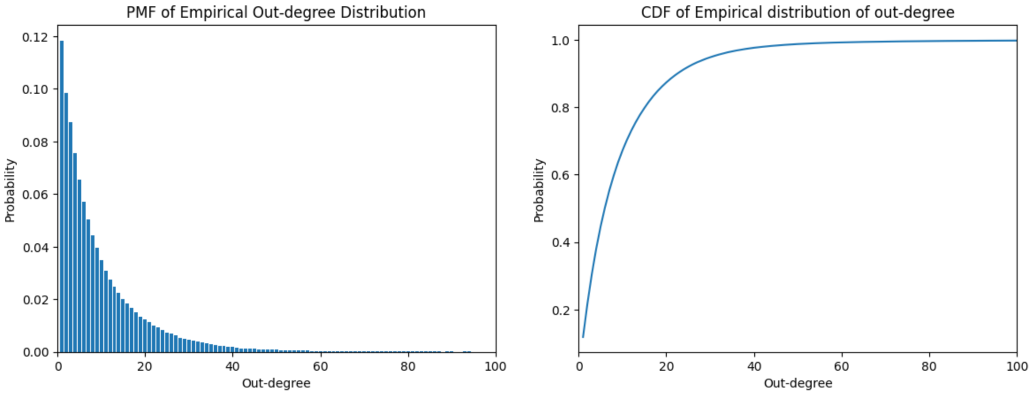


Figure A.4: Empirical Out-degree Distribution

| | |
|---|---|
| $\mathbb{E}[M]$ | 10.29 |
| $\text{Var}[M]$ | 181.189 |
| Median | 6 |
| Mode | 1 |
| Min | 1 |
| Max | 800 |

Table A.1: Descriptive statistics for empirical out-degree $M$

## A.3 Supercriticality of standard CTBP

In this section we will derive the supercriticality conditions for all combinations of mechanisms in the standard CTBP with rate given by (1). Let $\xi(t)$, $t > 0$, be the arrival process of offspring of a vertex in such CTBP. A supercritical CTBP grows approximately as $e^{\alpha t}$, where $\alpha > 0$ is the so-called *Malthusian* parameter. Formally, a CTBP is supercritical if there exists $\alpha > 0$ such that

$$\int_0^\infty e^{-\alpha t}\mathbb{E}\left[\xi(dt)\right] = 1. \tag{A.1}$$

The point process $\xi(t)$ counts the number of offspring of the root vertex. We rewrite the left-hand side of (A.1) using integration by parts, as follows

$$\int_0^\infty e^{-\alpha t}\mathbb{E}\left[\xi(dt)\right] = \int_0^\infty \alpha e^{-\alpha t}\mathbb{E}\left[\xi(t)\right]\, dt = \mathbb{E}\left[\xi(X)\right],$$

where $X$ is an exponential random variable with parameter $\alpha$. It follows that $\alpha$ in (A.1) exists if and only if $\mathbb{E}\left[\xi(\infty)\right] > 1$. The supercriticality condition is then

$$\mathbb{E}\left[\xi(\infty)\right] > 1. \tag{A.2}$$

We will now apply (A.2) to our nine models:

[U ] The rate of producing offspring at age $t > 0$ is 1, therefore $\mathbb{E}\left[\xi(\infty)\right] = \infty$, the CTBP is supercritical.

[P ] The rate of producing offspring at age $t > 0$ is $a\xi(t) + b$. We have that $\mathbb{E}\left[\xi(\infty)\right] = \infty$ if $b > 0$ and $\xi(\infty) = 0$ otherwise. The supercriticality condition is therefore: $b > 0$.

[$\text{F}_{\text{PL}}$, $\text{F}_{\text{EXP}}$ ] The rate of producing offspring is $\eta$. Therefore, both exponential and power law fitness result in $\mathbb{E}\left[\xi(\infty)\right] = \infty$, the corresponding CTBP's are supercritical.

[$\text{F}_{\text{PL}}\text{A}$ ] The rate of producing offspring is $\eta h(t)$, and the total number of offspring is $\eta H(\infty) = \eta$, where $H(t)$ is the cumulative aging function given by $H(t) = \int_0^t h(s)ds$. Therefore, the supercriticality condition is

$$\mathbb{E}\left[\eta\right] = \frac{(\tau-1)x_{\min}}{\tau-2} > 1.$$

[$\text{F}_{\text{EXP}}\text{A}$ ] Similarly as in $\text{F}_{\text{PL}}\text{A}$, the supercriticality condition is

$$\mathbb{E}\left[\eta\right] = \lambda^{-1} > 1.$$

[$F_{\text{UNIF}}$P ] The rate of producing offspring is $\eta(a\xi(t)+b)$. The supercriticality condition is:

$$b > 0, \quad P(\eta > 0) > 0.$$

[AP ] The rate of producing offspring at age $t > 0$ is $h(t)(a\xi(t)+b)$. By solving the differential equation $y'(t) = h(t)(ay(t)+b)$ with initial conditions $y(0) = 0$ and $y'(0) = h(0)b$, we obtain that the average number of offspring produced by time $t$ is $\frac{b}{a}e^{aH(t)} - \frac{b}{a}$. Since $H(\infty) = 1$, we obtain the supercriticality condition

$$\frac{b}{a}e^{a} - \frac{b}{a} > 1.$$

[$F_{\text{EXP}}$AP ] The rate of producing offspring is $\eta h(t)(a\xi(t)+b)$. By solving the differential equation $y'(t) = \eta h(t)(ay(t)+b)$ with initial conditions $y(0) = 0$ and $y'(0) = \eta h(0)b$, we obtain that the average number of offspring produced by time $t$ is $\frac{b}{a}e^{a\eta H(t)} - \frac{b}{a}$. Using again that $H(\infty) = 1$, we obtain the supercriticality conditions:

$$\frac{b}{a}\mathbb{E}\left[e^{\eta a}\right] - \frac{b}{a} > 1.$$

Recall that $\eta$ has exponential distribution with parameter $\lambda$. If $\lambda < a$, then the left-hand side is infinite, so the process is supercritical. If $\lambda > a$, then the condition becomes

$$\frac{b}{\lambda - a} > 1.$$

[A ] The model with only aging is excluded from our analysis because in our chosen setting this model is not supercritical. Indeed, our assumption that $h$ is the log-normal density, we have that $\mathbb{E}[\xi(\infty)] = 1$.

## A.4 Supercriticality of collapsed CTBP

The collapsing procedure that we propose in this paper is motivated by applications in citation networks, and does not permit easy derivation of supercriticality conditions. When $M = m$ is a constant, and the birth times of the vertices in CTBP are not adjusted, the supercriticality condition becomes

$$\frac{1}{m}\mathbb{E}[\xi(\infty)] > 1. \tag{A.3}$$

When $M$ is random, a batch of $M$ vertices born one after another receives factor $1/M$ to their rate of producing offspring. Therefore, the probability that a randomly sampled vertex in CTBP has factor $1/m$, is $\frac{mP(M=m)}{\mathbb{E}[M]}$ as in the size-biased distribution of $M$. Hence, on average, this factor is

$$\sum_m \frac{1}{m} \cdot \frac{mP(M=m)}{\mathbb{E}[M]} = \frac{1}{\mathbb{E}[M]}.$$

Yet, we cannot simply replace constant $m$ by $\mathbb{E}[M]$ in (A.3) for two reasons. First, the factor $1/M$ is assigned to a batch of consecutive vertices of CTBP, and not independently to each vertex. The difference is considerable because, if the factor $1/M$ was sampled independently from the size-biased distribution, then large values of $M$ had a high chance to appear quickly, slowing down the growth of CTBP due to the factor $1/M$, so this makes extinction more likely. On the other hand, in our procedure, we sample from the distribution of $M$ for each batch, so large values of $M$ are likely to appear later, and before they appear, the CTBP might already generate many offspring to ensure survival. At this point, we cannot exactly quantify the effect of large values of $M$ appearing independently for each vertex in CTBP or in batches. Second, and maybe even more importantly, we assign earlier birth times to the vertices in CTBP that create a collapsed vertex $v$. Then, many vertices of CTBP start generating offspring earlier than they would originally, which increases the chance of survival. Again, there are no analytical results that evaluate the effect of earlier birth times on the survival of the CTBP.

In our experiments we always chose parameters that satisfy (A.2), because the collapsed process can survive only if the CTBP with $M = 1$ survives. Further, we found that the collapsed CTBP process often survives even when (A.3) is violated, rendering requirement (A.3) conservative. As an example, Figure A.5 shows survival and extinction of the collapsed process for the model $F_{\text{PL}}$A. The figure shows the number of simulations that died out for $\tau$ between 2 and 2.7, with $x_{\min}$ sampled randomly from the interval $(0.5, 1)$ as stated in Table 3. When $\tau = 2.7$, the value $\frac{(\tau-1)x_{\min}}{\tau-2}$ computed in Section A.3, ranges from 1.2143 to 2.4286, which is much smaller than $\mathbb{E}[M] = 10.29$. In Figure A.5, when $\tau$ grows, we see a slight increase in the number of outliers, but all choices of $\tau$ still succeed in under 20 attempts, with the vast majority succeeding in less than 3.

As a final note, the model $F_{\text{EXP}}$AP has many parameters, so we use (A.3) to define the range of $\lambda$.

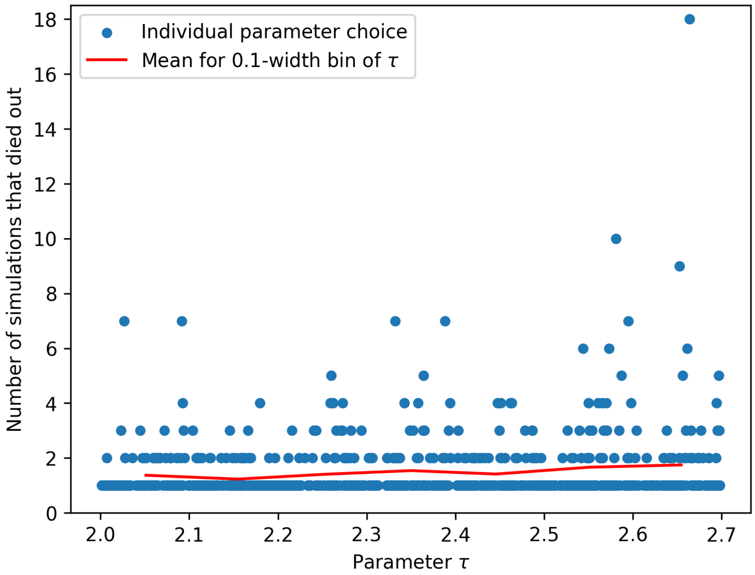


Figure A.5: Number of attempts for simulation of class $F_{PL}A$ that died out.

## A.5 Power-law degree distribution in F and FA models

**Proposition A.1** *Consider a supercritical CTBP without preferential attachment, and let $(p_k)$ be its limiting degree distribution. Suppose that the fitness of each vertex is an independent copy of random variable $\eta$ with $\mathbb{E}[\eta] < \infty$, and let $\gamma$ be an aging random variable with density $h$. Then the following statements hold:*

*(i) If $\eta$ has a power-law distribution, then $p_k$ decays as a power law with the same exponent.*

*(ii) If $\eta$ has an exponential distribution, then $p_k$ decays faster than any power law.*

*Proof.* Let $X$ be an exponentially distributed random variable with parameter $\alpha$ that is the Malthusian parameter of the supercritical CTBP. We will use the fact that $p_k$ equals the probability mass function of the number of offspring of the root vertex of the CTBP at time $X$ [GHW17, Definition 3.12]. Since the root produces children according to a Poisson process, we have that

$$p_k = \mathbb{E}\left[e^{-\gamma\eta X}\frac{(\gamma\eta X)^k}{k!}\right]. \tag{A.4}$$

In (A.4), the main contribution comes from $\gamma\eta X = k(1 + o(1))$ as $k \to \infty$. Thus, the decay of $\sum_{s=k}^{\infty} p_s$ is the same as that of $\mathbb{P}(\gamma\eta X \geq k)$. Since $X$ and $\gamma$ have all finite moments, and $\gamma$, $\eta$, $X$ are independent, the power law of $\eta$ implies the power law of $\gamma\eta X$ with the same exponent as $\eta$ (see e.g. [DM08, Lemma 2.3]). This proves (i). Next, if $\eta$ has exponential distribution, then $\gamma\eta X$ has all finite moments and thus $\mathbb{P}(\gamma\eta X \geq k)$ has no power-law decay, which proves (ii). □

## A.6 Size and number of networks in the synthetic dataset

**Choosing the size of synthetic networks.** We aim at synthetic networks' size of the order $10^4$ because our real-life WoS dataset has networks ranging from $80,000$ to $400,000$ papers. Generating the networks, especially with aging, is computationally expensive, therefore, the networks' size shouldn't be larger than necessary. Our choice for the size $20,000$ is justified as follows. First, we generate a small dataset, 40 networks for each category, with $20,000$ vertices. Then, we calculate the time-cohort feature matrix for this dataset at various sizes of the network: $\{1 \cdot 10^3, \ldots, 20 \cdot 10^3\}$. We then plot the *learning curve*, see Figure A.6a. The plot shows the mean accuracy of 100 decision trees with random 80-20 train-test splits, as a function of the networks' size. We use a decision tree as a baseline for the amount of signal in the features. The classification accuracy continues to improve as the network grows, but levels off after the networks' size $10,000$. From this, we conclude that the network size $20,000$ in the synthetic dataset is sufficient for our classification task.

**Choosing the number of synthetic networks.** Our final synthetic dataset consists of 6733 networks of size $20,000$, with 750 or slightly less networks for each of the nine models (17 parameter combinations in models with aging repeatedly resulted in extinction). To determine whether the dataset is large enough, we plot a learning curve over the training set size, see Figure A.6b. Specifically, we vary the size of the training set, and

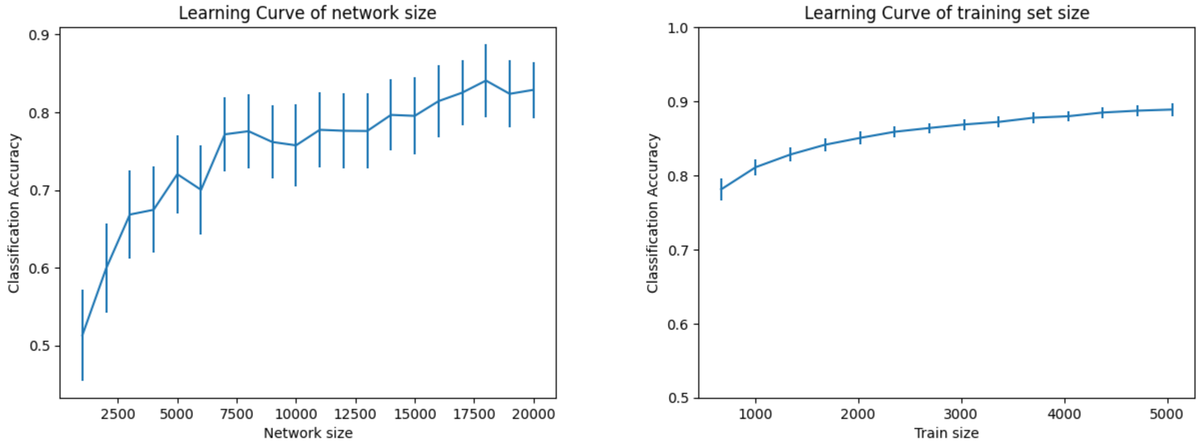


(a) (b)

Figure A.6: Learning curves over: (a) the size of a network in the synthetic dataset; (b) the number of synthetic networks in the training set.

use the rest of synthetic networks in the test set. For each training set size, we train 100 decision trees with 100 different train-test splits, and consider the classification accuracy. As before, the decision tree classifier was chosen merely as a metric for the amount of signal in the data. We observe that the learning curve flattens well before the largest training set size. We conclude that we have sufficient synthetic data.

**Computational resources.** The collapsed CTBP simulation was implemented in Python. The generation of 6733 networks took 20 hours, using all cores on an Intel i7-7700HQ.[1]

## A.7 Choosing a classifier

We have classified synthetic dynamic networks in one of the nine categories (each category corresponding to one generative model), using state-of-the-art static features, our novel dynamic features, and the combination thereof. We considered a broad variety of classifiers with their scikit-learn implementation [Ped+11]: support vector classifiers, tree-based models (decision tree), Naive Bayes (Gausian, Multinomial), and ensemble models (Random Forest, Histogram-based Gradient Boosting Classification Tree). Performance is evaluated in terms of total classification accuracy and a confusion matrix. We take the standard 20-80 stratified test-train split, to ensure that both the test and train set are balanced for each category. We use 5-fold cross validation on the training set to select the classification model and tune hyperparameters. We obtain the best performance in the cross validation with a state-of-the-art Histogram-based Gradient Boosting Classification Tree with the logistic loss function. The final accuracy and confusion matrices for the classification of the synthetic data in Section 2.3, are on the test set. For the classification of citation networks in Section 2.4, we train the classifier on the full synthetic data set.

## A.8 Feature importance analysis

Figure A.7 presents the permutation importance results. Among static features, in Figure A.7(a),the standard deviation of the clustering coefficient comes out as most distinctive feature. Assortativity comes in third. In the future, this should be verified on networks of different sizes as assortativity can be size-dependent in power-law networks [LV13]. The low values of the permutation importance suggest that none of the features dominates the performance. We did not study the redundancy among static features any further because we use them merely as a benchmark from the literature. In Figure A.7(b),(c), the total permutation importance of the top features (dynamic features in Figure A.7(b), and the combination of static and dynamic features in Figure A.7(c)) turns out to be much smaller than 1. This renders permutation importance uninformative. Indeed, the permutation importance of a feature equals to the decrease in performance when this feature is randomly permuted over the dataset. When the sum of permutation importance scores is small, it means that there are correlations between the features: if information carried by one feature is removed due to the random permutation, then other features supply a similar information to the classifier, so the performance decrease is small.

[1] https://gitfront.io/r/user-6239985/R9WcT8Msr46T/DynamicNetworkSimulation/

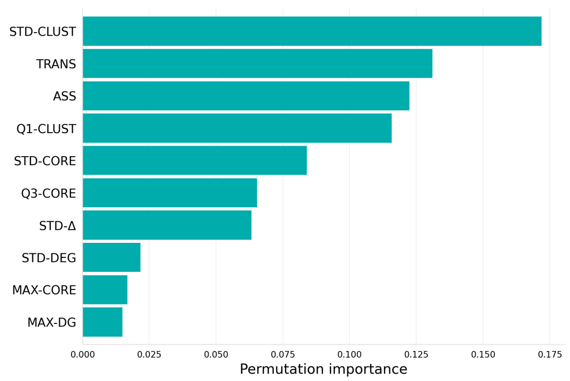


(a)

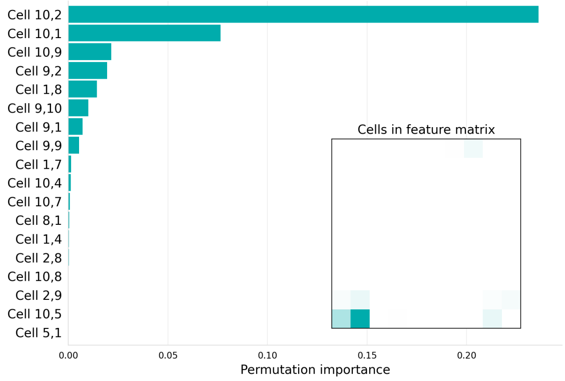


(b)

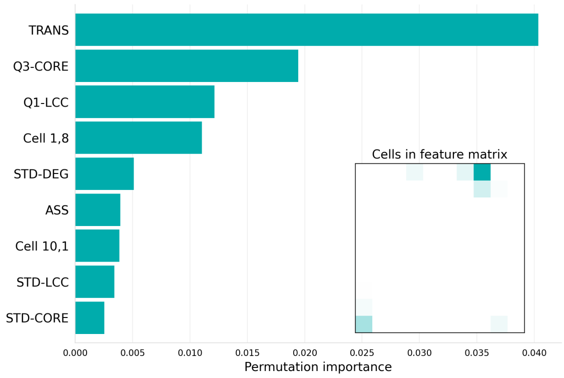


(c)

Figure A.7: Permutation importance of: (a) static features, (b) dynamic features with time-cohorts, (c) both dynamic and static features with time-cohorts. Abbreviations for the static features are as follows. STD-CLUST: standard deviation of clustering coefficient; TRANS: transitivity (in the giant connected component); ASS: Assortativity; Q1-CLUST: first quantile of clustering coefficient; STD-CORE: standard deviation of coreness, Q3-CORE: third quantile of coreness; STD-Δ: standard deviation of triangles; STD-DEG: standard deviation of degree, MAX-CORE: maximum coreness, MAX-DEG: maximum degree; Q1- and STD-LCC: 1st quantile and resp. standard deviation of local clustering coefficient.

## A.9 Size-cohort dynamic feature matrix

Here, we report on the performance of the classifier trained on the *size*-cohort feature matrix, i.e. each row is computed based on the arrival of an equal number of new vertices (see Section 4.2). Recall that the main text presents the results for the feature matrix with *time*-cohorts, where the rows are computed based on the arrivals in equal time intervals. The size-cohort dynamic features perform comparable to the time-cohort dynamic features, yielding an accuracy of 97.32%, see the confusion matrix in Figure A.8(a). The feature importance in Figure A.8(b) again has sum of the scores much lower than one, therefore, is not informative. When training on both static and size-cohort dynamic features, we get the 97.62% accuracy, see the confusion matrix in figure A.8(c). Permutation importance, presented in Figure A.8(d), is again uninformative.

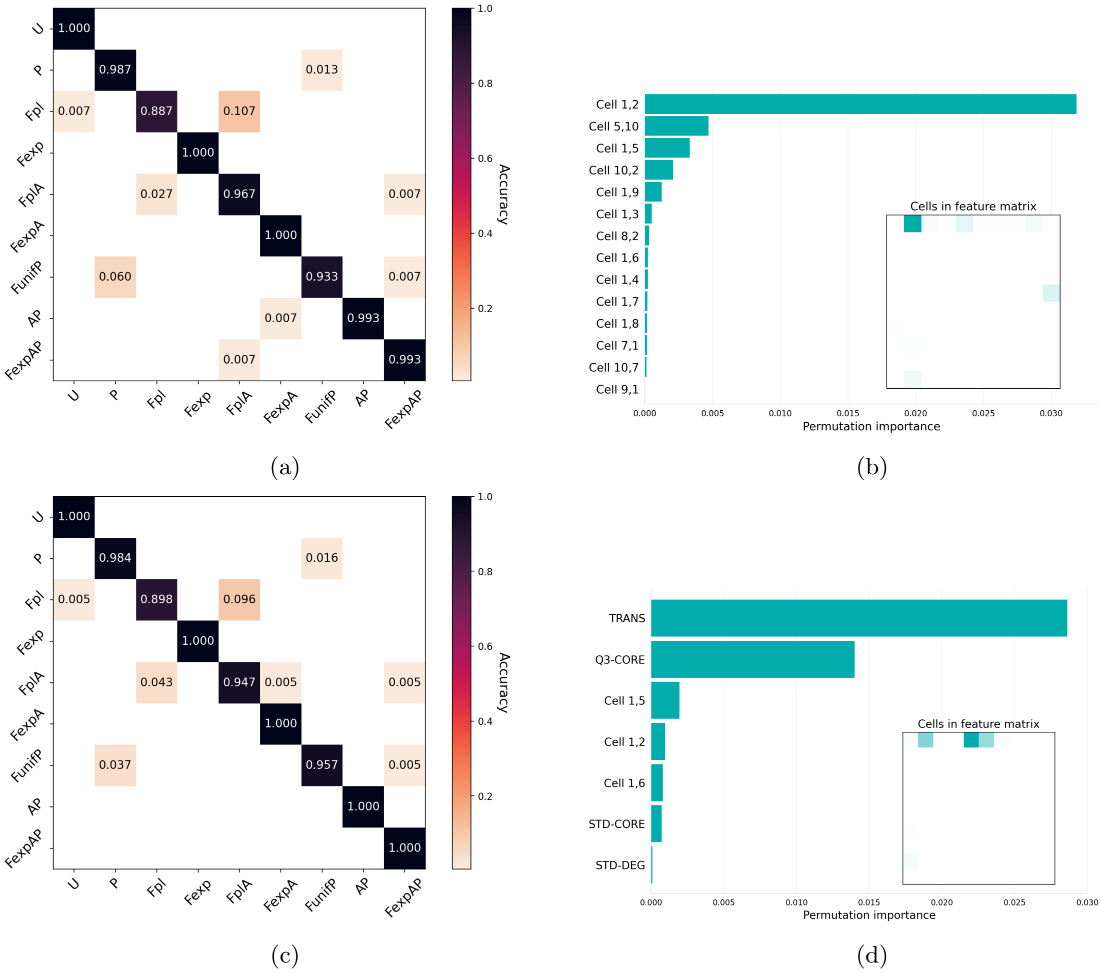


Figure A.8: (a) Confusion matrix using size-cohort dynamic feature matrix, (b) Feature permutation of the size-cohort dynamic feature matrix, (c) Confusion matrix using both static and size-cohort dynamic features, (d) Feature permutation of both dynamic and static features. TRANS: transitivity (gcc), Q3-CORE: 3rd quantile of Core, STD-CORE: standard deviation of core, STD-DEG: standard deviation of degree.

## A.10 UMAP Visualization of Features and Citation Networks

In search for an interpretable explanation of why different features may place the same citation network in different classes, we created a two-dimensional embedding of the features using UMAP (Uniform Manifold Approximation and Projection), a method for general non-linear dimension reduction [MHM20]. UMAP can be seen as an unsupervised classification method based on only two features derived from the vector of all features. Figure A.9 shows the two-dimension UMAP embedding of four feature combinations for the synthetic networks. The same embedding is applied to feature combinations of the citation networks, and they are overlayed in the visualization. We see that UMAP separates some of the classes, thus showing that the features presented in this paper can be used for unsupervised model selection of dynamic networks. However, a striking observation is that there is a significant overlap between classes of synthetic networks. This underlines the complexity of the problem. Indeed, even for synthetic networks, a two-dimensional embedding is not able to identify the differences between some classes. We also see that the placement of citation networks in the embedding space is not consistent with the supervised machine learning classifications (see Table 2). However, given the large overlap of the classes, this placement is not reliable and only confirms the sensitivity of classification to chosen features. We conclude that there is no simple explanation why different features classify citation networks differently. The most likely explanation is that none of the models accurately describes all aspects of citation networks. Then, different features capture different aspects of real-life networks, and thus may produce a different label. The experiments with UMAP embedding confirm our cautionary tale that applying machine learned model selection in practice requires significant care in justification and validation of feature design.

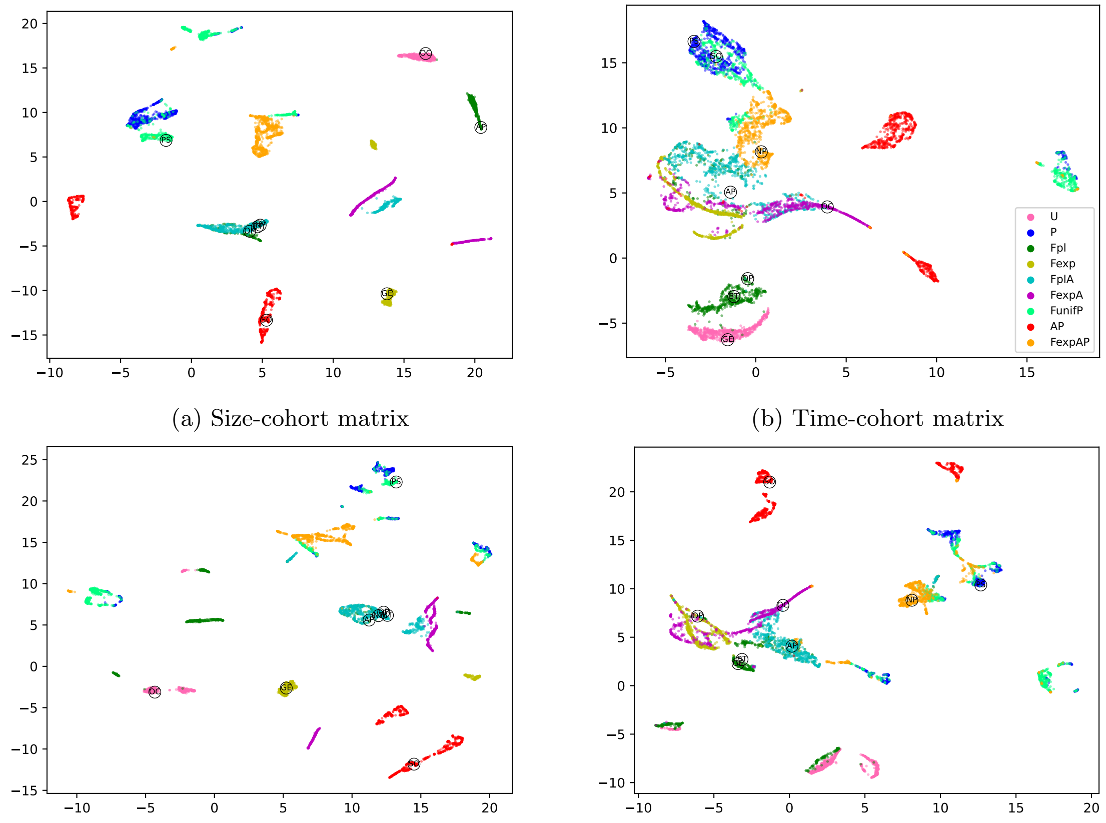


(a) Size-cohort matrix

(b) Time-cohort matrix

(c) Size-cohort matrix + static features

(d) Time-cohort matrix + static features

Figure A.9: UMAP embedding in two dimensions for feature combinations. Citation networks denoted by black circle with field acronym.

## A.11 Citation network classifications

In Section 2.4 we have reported the most likely model for each real-life network. In Tables A.2– A.6 below we present the entire distribution over the models for each citation network and all combinations of the features. The column **'Prediction'** states the most likely model.

| WoS Field | Prediction | U | P | $F_{PL}$ | $F_{EXP}$ | $F_{PL}A$ | $F_{EXP}A$ | $F_{UNIF}P$ | AP | $F_{EXP}AP$ |
|---|---|---|---|---|---|---|---|---|---|---|
| AP | $F_{EXP}A$ | 10.194% | 0.0098% | 0.8629% | 0.0087% | 1.7351% | **87.1539%** | 0.0302% | 0.0033% | 0.0022% |
| BT | AP | 0.0054% | 0.2101% | 0.0042% | 0.017% | 1.6216% | 7.7802% | 0.0047% | **83.0959%** | 7.2608% |
| GE | AP | 0.0004% | 0.0074% | 0.0002% | 0.0007% | 0.1266% | 0.1152% | 0.0002% | **98.8932%** | 0.8562% |
| NP | $F_{EXP}A$ | 0.008% | 1.3284% | 0.0437% | 0.022% | 2.0946% | **91.7197%** | 0.0034% | 4.6799% | 0.1002% |
| OC | $F_{EXP}A$ | 0.3216% | 48.8172% | 0.2431% | 0.0114% | 1.0426% | **48.9337%** | 0.0134% | 0.544% | 0.0732% |
| OP | AP | 0.0033% | 0.353% | 0.0026% | 0.0088% | 0.241% | 14.5056% | 0.0015% | **81.4642%** | 3.4201% |
| PS | AP | 0.005% | 0.2505% | 0.0088% | 0.0196% | 3.5427% | 14.1684% | 0.0066% | **72.324%** | 9.6744% |
| SO | AP | 0.0007% | 0.0437% | 0.0009% | 0.0034% | 0.2198% | 0.5241% | 0.0009% | **95.7194%** | 3.4872% |

Table A.2: Class probabilities for citation networks classified using only static features.

| WoS Field | Prediction | U | P | $F_{PL}$ | $F_{EXP}$ | $F_{PL}A$ | $F_{EXP}A$ | $F_{UNIF}P$ | AP | $F_{EXP}AP$ |
|---|---|---|---|---|---|---|---|---|---|---|
| AP | AP | 0.0001% | 0.0001% | 0.0002% | 0.0001% | 0.0008% | 0.6312% | 0.0001% | **99.3624%** | 0.0048% |
| BT | AP | 0.0001% | 0.0001% | 0.0002% | 0.0001% | 0.0005% | 0.1622% | 0.0001% | **99.7829%** | 0.054% |
| GE | AP | 0.0002% | 0.0001% | 0.0004% | 0.0002% | 0.0032% | 0.5906% | 0.0004% | **99.4024%** | 0.0025% |
| NP | AP | 0.0002% | 0.0002% | 0.0004% | 0.0002% | 0.0012% | 0.6091% | 0.0002% | **96.7235%** | 2.665% |
| OC | AP | 0.0001% | 0.0001% | 0.0003% | 0.0001% | 0.0008% | 0.245% | 0.0001% | **99.5958%** | 0.1576% |
| OP | AP | 0.0001% | 0.0% | 0.0001% | 0.0001% | 0.0012% | 0.1917% | 0.0001% | **99.7396%** | 0.0671% |
| PS | AP | 0.0013% | 0.0017% | 0.0093% | 0.0015% | 0.0082% | 1.5193% | 0.0012% | **95.0033%** | 3.4543% |
| SO | AP | 0.0002% | 0.0002% | 0.0012% | 0.0002% | 0.001% | 0.4056% | 0.0005% | **99.578%** | 0.0131% |

Table A.3: Class probabilities for citation networks classified using only time-cohort dynamic features.

| WoS Field | Prediction | U | P | $F_{PL}$ | $F_{EXP}$ | $F_{PL}A$ | $F_{EXP}A$ | $F_{UNIF}P$ | AP | $F_{EXP}AP$ |
|---|---|---|---|---|---|---|---|---|---|---|
| AP | $F_{EXP}AP$ | 0.006% | 0.0009% | 0.0041% | 0.0176% | 0.0127% | 0.0026% | 6.1683% | 3.3167% | **90.4711%** |
| BT | $F_{EXP}AP$ | 0.0203% | 0.0005% | 0.0048% | 0.0064% | 0.0384% | 0.0949% | 7.2838% | 0.6714% | **91.8795%** |
| GE | $F_{EXP}AP$ | 0.1216% | 0.0174% | 0.0552% | 0.3496% | 0.1957% | 1.4084% | 14.3512% | 4.8238% | **78.6771%** |
| NP | $F_{EXP}AP$ | 0.0078% | 0.0006% | 0.0732% | 0.0069% | 0.1857% | 0.0481% | 0.87% | 1.1023% | **97.7054%** |
| OC | $F_{EXP}AP$ | 12.6796% | 0.0009% | 0.0018% | 0.0209% | 0.0026% | 0.0027% | 17.0127% | 4.1622% | **66.1166%** |
| OP | $F_{EXP}AP$ | 0.0038% | 0.0016% | 0.0035% | 0.0079% | 0.0335% | 0.0862% | 10.7796% | 0.6463% | **88.4376%** |
| PS | $F_{EXP}AP$ | 0.0002% | 0.0005% | 0.0006% | 0.0022% | 0.0021% | 0.0176% | 1.1467% | 0.0716% | **98.7585%** |
| SO | P | 0.8341% | **49.6114%** | 0.0571% | 1.0126% | 0.3739% | 1.2897% | 8.8134% | 12.6348% | 25.373% |

Table A.4: Class probabilities for citation networks classified using only size-cohort dynamic features.

| WoS Field | Prediction | U | P | $F_{PL}$ | $F_{EXP}$ | $F_{PL}A$ | $F_{EXP}A$ | $F_{UNIF}P$ | AP | $F_{EXP}AP$ |
|---|---|---|---|---|---|---|---|---|---|---|
| AP | $F_{EXP}A$ | 0.0295% | 0.01% | 0.1138% | 0.0525% | 4.6638% | **94.2683%** | 0.0238% | 0.6141% | 0.2241% |
| BT | $F_{EXP}AP$ | 0.0061% | 0.0091% | 0.0372% | 0.0278% | 0.08% | 1.4155% | 0.0081% | 36.3496% | **62.0666%** |
| GE | AP | 0.0005% | 0.0001% | 0.0002% | 0.0002% | 0.0009% | 0.0167% | 0.0001% | **99.8106%** | 0.1707% |
| NP | $F_{EXP}A$ | 0.0069% | 0.0086% | 0.0436% | 0.0399% | 0.1774% | **59.7829%** | 0.0062% | 8.1347% | 31.7999% |
| OC | $F_{EXP}A$ | 0.272% | 0.053% | 0.2732% | 0.0929% | 0.2399% | **73.6001%** | 0.0391% | 11.526% | 13.9039% |
| OP | $F_{EXP}AP$ | 0.005% | 0.0061% | 0.0274% | 0.0278% | 0.0862% | 10.2059% | 0.0047% | 29.6566% | **59.9803%** |
| PS | $F_{EXP}AP$ | 0.0053% | 0.0065% | 0.029% | 0.0266% | 0.101% | 7.5905% | 0.0048% | 21.7334% | **70.5028%** |
| SO | AP | 0.1384% | 0.0011% | 0.0032% | 0.0074% | 0.0191% | 0.2522% | 0.0048% | **50.9388%** | 48.635% |

Table A.5: Class probabilities for citation networks classified using both time-cohort dynamic features and static features.

| WoS Field | Prediction | U | P | $F_{PL}$ | $F_{EXP}$ | $F_{PL}A$ | $F_{EXP}A$ | $F_{UNIF}P$ | AP | $F_{EXP}AP$ |
|---|---|---|---|---|---|---|---|---|---|---|
| AP | $F_{EXP}AP$ | 8.2686% | 0.3277% | 5.3107% | 9.5221% | 1.2493% | 5.9404% | 20.9093% | 0.8591% | **47.6128%** |
| BT | $F_{EXP}AP$ | 0.0667% | 0.0009% | 0.0185% | 0.0503% | 0.0077% | 0.0955% | 0.0426% | 21.8597% | **77.858%** |
| GE | AP | 0.0011% | 0.0001% | 0.0033% | 0.0048% | 0.0004% | 0.0015% | 0.0034% | **99.7651%** | 0.2204% |
| NP | $F_{EXP}AP$ | 0.0127% | 0.0005% | 0.0077% | 0.0082% | 0.0048% | 0.1248% | 0.0097% | 7.2476% | **92.584%** |
| OC | $F_{EXP}AP$ | 47.4841% | 0.0018% | 0.0324% | 0.0351% | 0.0042% | 0.1073% | 0.9745% | 0.7513% | **50.6094%** |
| OP | $F_{EXP}AP$ | 0.0165% | 0.003% | 0.0165% | 0.0554% | 0.0118% | 0.0985% | 0.0709% | 23.5339% | **76.1935%** |
| PS | $F_{EXP}AP$ | 0.0027% | 0.0026% | 0.0155% | 0.1971% | 0.0217% | 0.0442% | 0.0165% | 10.0808% | **89.6187%** |
| SO | AP | 0.1376% | 1.2988% | 0.0141% | 0.4178% | 0.0046% | 4.9587% | 0.0125% | **88.0893%** | 5.0665% |

Table A.6: Class probabilities for citation networks classified using both size-cohort dynamic features and static features.

## A.12 Correlations in Dynamic Feature matrix

Within the dynamic feature matrix there is correlation between cells. In Figure A.10 and Figure A.11 we show the correlations between the corner-cells and the other cells in the feature matrix, across all networks in the dataset. We see that cells adjacent to the corners are more stronger correlated, especially in top corners that correspond to the number of edges received from early cohorts. This suggests redundancy in the features. Nevertheless, in the experiments, excluding some cells from the feature matrix leads to an immediate decrease in classification accuracy. Indeed, Figures A.10, A.11 don't show any clear pattern that can be helpful for excluding some of the features.

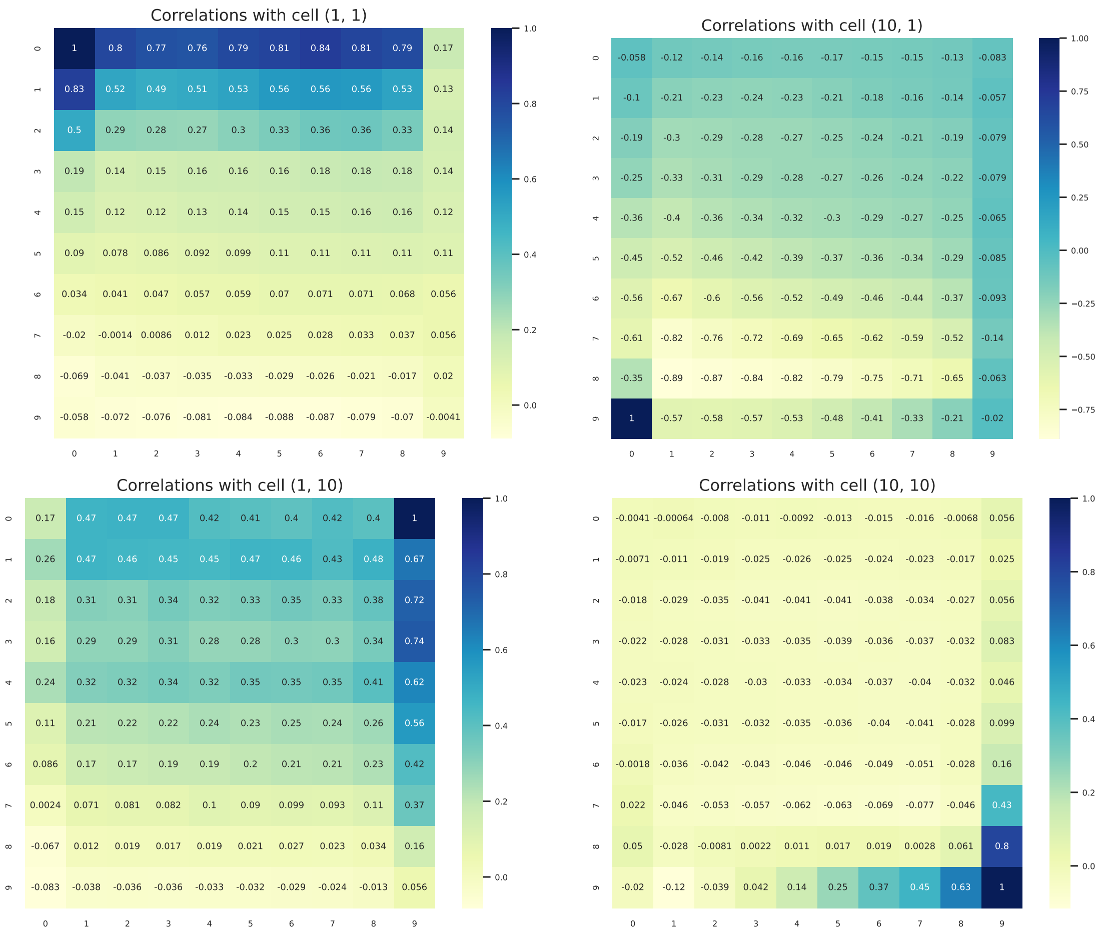


Figure A.10: Correlations in time-cohort dynamic feature matrix

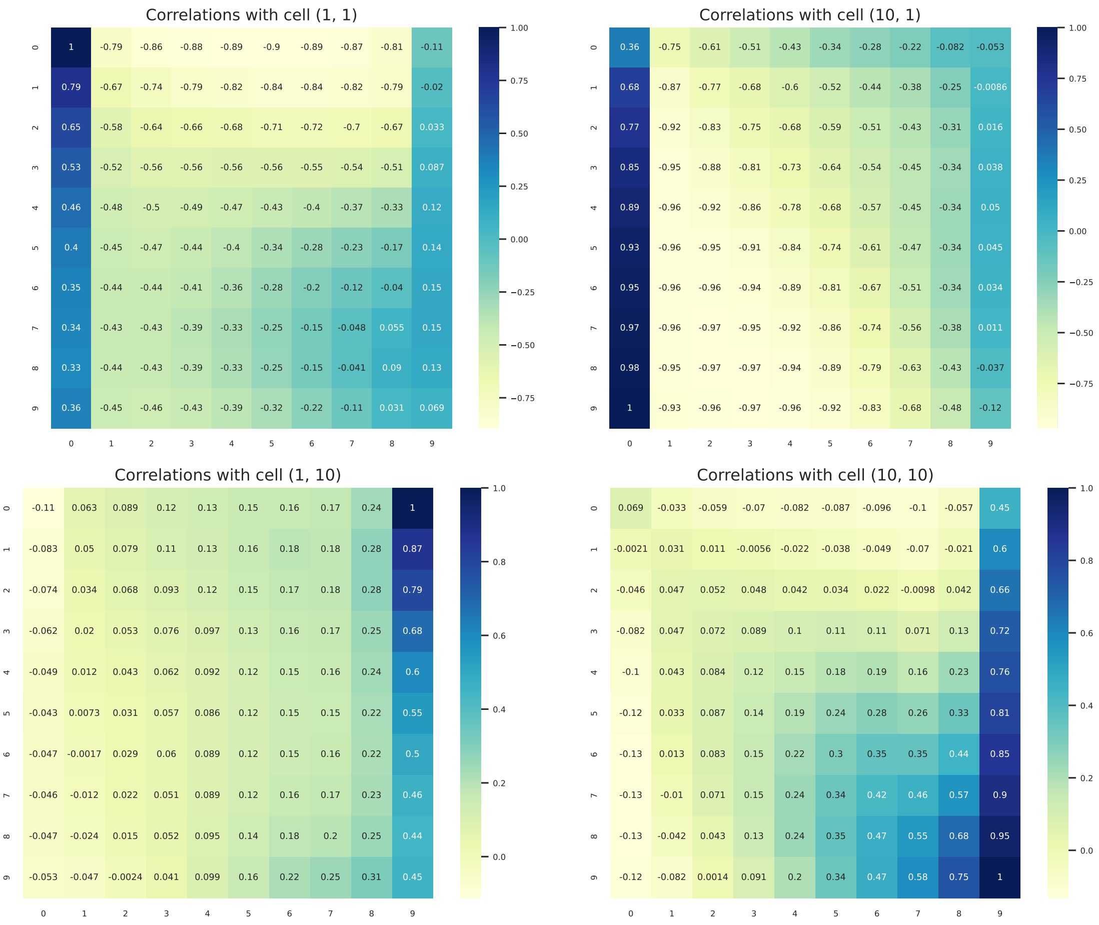


Figure A.11: Correlations in size-cohort dynamic feature matrix

# References

[Art+20] I. Artico et al. "How rare are power-law networks really?" In: *Proceedings of the Royal Society A* **476**.2241 (2020), p. 20190742.

[BC19] A. Broido and A. Clauset. "Scale-free networks are rare". In: *Nature Communications* **10**.1 (2019), p. 1017.

[DM08] Richard A Davis and Thomas Mikosch. "Extreme value theory for space–time processes with heavy-tailed distributions". In: *Stochastic Processes and their Applications* 118.4 (2008), pp. 560–584.

[GHW17] A. Garavaglia, R. van der Hofstad, and G. Woeginger. "The dynamics of power laws: fitness and aging in preferential attachment trees". In: *J. Stat. Phys.* **168**.6 (2017), pp. 1137–1179. ISSN: 0022-4715.

[Hof23] Remco van der Hofstad. *Random graphs and complex networks*. Vol. 2. To appear. 2023. URL: `https://www.win.tue.nl/~rhofstad/`.

[Hol19] P. Holme. "Rare and everywhere: Perspectives on scale-free networks". In: *Nature Communications* **10**.1 (2019), p. 1016.

[LV13] Nelly Litvak and Remco Van Der Hofstad. "Uncovering disassortativity in large scale-free networks". In: *Physical Review E* 87.2 (2013), p. 022801.

[MHM20] Leland McInnes, John Healy, and James Melville. *UMAP: Uniform Manifold Approximation and Projection for Dimension Reduction*. 2020. arXiv: `1802.03426 [stat.ML]`.

[Ped+11] Fabian Pedregosa et al. "Scikit-learn: Machine learning in Python". In: *Journal of machine learning research* 12.Oct (2011), pp. 2825–2830.

[Pri65] D. J. de Solla Price. "Networks of Scientific Papers". In: *Science* **149** (1965), pp. 510–515.

[Pri86] D. J. de Solla Price. *Little science, big science... and beyond*. Columbia University Press New York, 1986.

[Voi+19] I. Voitalov et al. "Scale-free Networks Well Done". In: *Physical Review Research* **1**.3 (2019), p. 033034.

[WMH14] J. Wang, Y. Mei, and D. Hicks. “Comment on ”Quantifying long-term scientific impact””. In: *Science* **345**.6193 (2014), p. 149. ISSN: 1095-9203.

[WSB13] D. Wang, C. Song, and A. L. Barabási. “Quantifying Long-Term Scientific Impact”. In: *Science* **342**.6154 (2013), pp. 127–132.

[WYY08] M. Wang, G. Yu, and D. Yu. “Measuring the preferential attachment mechanism in citation networks”. In: *Physica A: Statistical Mechanics and its Applications* **387**.18 (2008), pp. 4692–4698.
